# Stability of Patches of Higher Population Density within the Heterogenous Distribution of the Gray Field Slug *Deroceras reticulatum* in Arable Fields in the UK

**DOI:** 10.3390/insects12010009

**Published:** 2020-12-25

**Authors:** Emily Forbes, Matthew Back, Andrew Brooks, Natalia B. Petrovskaya, Sergei V. Petrovskii, Tom Pope, Keith F.A. Walters

**Affiliations:** 1 Centre for Integrated Pest Management, Harper Adams University, Shropshire TF10 8NB, UK; eforbes@harper-adams.ac.uk (E.F.); mback@harper-adams.ac.uk (M.B.); Asbrooks@harper-adams.ac.uk (A.B.); tpope@harper-adams.ac.uk (T.P.); 2 School of Mathematics, University of Birmingham, Birmingham B15 2TS, UK; n.b.petrovskaya@bham.ac.uk; 3 School of Mathematics and Actuarial Science, University of Leicester, Leicester LE1 7RH, UK; sp237@leicester.ac.uk

**Keywords:** *Deroceras reticulatum*, aggregation, spatial clustering, spatial density distribution, spatio-temporal stability

## Abstract

**Simple Summary:**

There is growing public and legislative pressure to reduce the use of pesticides in crop production. It is thought that the gray field slug is not uniformly distributed in arable fields, but that patches of higher slug densities occur, interspersed within areas of lower slug numbers. Targeting molluscicide applications only at these patches, leaving other areas untreated, would substantially reduce the amount of molluscicide used but relies on patches being a feature of slug populations in all susceptible crops, and always occurring in the same places during the periods slug control is needed. This study investigated these requirements in 22 commercial arable field crops from different regions of the UK, and in three different years. Despite a variable proportion of slug populations being found below the soil surface (where they could not be assessed) on different sampling dates, existence of higher density patches were confirmed in all fields, although it was noted that, when too few slugs were active on the soil surface, they could not be reliably detected. When they were detected, they occurred in the same locations in each field. The potential for using these patches in more sustainable slug control approaches is discussed.

**Abstract:**

Exploitation of heterogenous distributions of *Deroceras reticulatum*, in arable fields by targeting molluscicide applications toward areas with higher slug densities, relies on these patches displaying sufficient spatio-temporal stability. Regular sampling of slug activity/distribution was undertaken using 1 ha rectangular grids of 100 refuge traps established in 22 commercial arable field crops. Activity varied significantly between the three years of the study, and the degree of aggregation (Taylor’s Power Law) was higher in fields with higher mean trap catches. Hot spot analysis detected statistically significant spatial clusters in all fields, and in 162 of the 167 individual assessment visits. The five assessment visits in which no clusters were detected coincided with low slug activity (≤0.07 per trap). Generalized Linear Models showed significant spatial stability of patches in 11 fields, with non-significant fields also characterized by low slug activity (≤1.2 per trap). Mantel’s permutation tests revealed a high degree of correlation between location of individual patches between sampling dates. It was concluded that patches of higher slug density were spatio-temporally stable, but detection using surface refuge traps (which rely on slug activity on the soil surface) was less reliable when adverse environmental conditions resulted in slugs retreating into the upper soil horizons.

## 1. Introduction

The gray field slug, *Deroceras reticulatum* (Müller), is a widespread and economically important pest in temperate regions throughout the world, damaging a wide range of field crops [1,2]. In the UK, slugs are considered to be one of the most damaging pests of arable crops and, in the absence of effective control, it is estimated that they would cost the industry up to GBP 100 million per year [3,4]. Management in most arable crops relies on conventional molluscicides applied as pellets to the soil surface and usage of these products fluctuates between years and regions in relation to environmental factors such as autumn rainfall. Average usage is usually high; however, for example, in 2018, a total of 471,872 ha wheat and 390,828 ha oilseed rape each received a mean of two applications of molluscicides, while 55,745 ha potatoes received between 2 and 3 applications (27, 67 and 39% of total crop areas respectively) [5]. A biological control agent for slugs (the nematode *Phasmarhabditis hermaphrodita*) is also available but, in the UK, use of this option is generally confined to high value horticultural crops and it is currently not cost effective in crops such as cereals or oilseed rape [6,7,8,9].

There is growing public and legislative pressure to reduce the use of conventional control agents in crop production, due to their contribution to air, soil and water pollution, and as a result of perceived risks of environmental damage or adverse effects on human health [10,11,12,13,14,15]. The limited number of active ingredients available for slug control in Europe was exacerbated by the loss of approval for Methiocarb in 2015, and Metaldehyde (currently the subject of national restrictions), requires re-approval in 2023 [4]. Use of the few available molluscicides on such large scales, therefore, make new approaches to sustainable use of the products a high priority.

The potential for reducing pesticide use on farms by spatially targeting applications to specific areas of arable fields to control pest and weeds has attracted repeated interest (e.g. [16,17,18,19]), with studies investigating a range of approaches including, amongst others, use of the probability of encountering target species in a spatial environment, development of field contour maps of defined thresholds, and automated robotic pesticide spraying over target areas [20,21,22]. *Deroceras reticulatum* is known to display a heterogenous distribution in arable crops, characterized by discrete patches of higher slug densities dispersed across the field with fewer slugs in intervening areas [11,23,24,25,26]. This may offer the potential for targeting molluscicide treatments only at higher density patches, but successful use of the technique relies on an understanding of several key factors, including the degree of spatio-temporal stability of the patches and the biological mechanisms underpinning patch cohesion. In addition, the range of patch sizes encountered and frequency of occurrence should facilitate the automation of targeted treatment across fields, and an approach incorporating action thresholds into a commercially viable management procedure for patch treatment is required. Such a procedure will also rely on the development of a commercially viable method of identifying patch location and dimensions.

Recent empirical studies conducted in the major crop growing regions of the UK have confirmed the existence of slug patches in commercial fields [24]. Populations of *D. reticulatum* are, however, distributed between the soil surface and the upper soil horizon, with a smaller proportion being active on the surface during periods of sub-optimal temperatures or dry weather [25]. The large but variable proportion that are located within the upper soil horizons cannot be easily detected using the standard surface refuge traps used in these experiments. Slug patches identified during periods with favourable conditions were, therefore, found to be difficult to locate during periods with adverse weather but re-appeared in the same locations as conditions improved, demonstrating potential temporal stability [24]. This conclusion was supported by studies of the movement of individual slugs in field crops, which have shown that most forage within a limited area [27]. Recent work has provided an insight into an underlying mechanism leading to this observation, by showing that components of slug movement (average velocities, turning angles and movement/resting times) vary between slugs in higher and lower density areas, resulting in them dispersing more slowly when in higher density patches of conspecifics [28]. Confirmation of the spatio-temporal stability of slug patches in arable crops, however, awaits detailed analysis of empirical field data from commercial crops in different cropping years. Modelling studies based on extensive empirical field data have also demonstrated that the range of patch sizes encountered and their frequency of occurrence permit the automation of targeted treatment across fields [29]. Threshold-based decision making for such treatments have also been addressed, demonstrating that the procedure would reduce pesticide applications by up to 40–50% in some fields, increasing the importance of confirmatory work on patch stability [29].

In this study, data collected from 22 commercial crops (6 crop types) in 4 arable regions of the UK, and from 3 successive cropping seasons were analyzed to investigate the degree of heterogeneity of *D. reticulatum* populations in each. The distribution of the slugs between sequential assessment dates was compared to test the hypothesis that the location of higher density patches within a field remains stable throughout the crop growing season.

## 2. Materials and Methods

Sampling was conducted in three consecutive growing seasons in commercial crops from major UK arable regions. Each crop was grown as part of rotations that included oilseed rape and wheat, and as position in a rotation can affect slug numbers [30], both the crop sampled and the preceding crop were recorded (Table 1). In all cases normal agronomic practices for the farm were followed. Work was conducted in 5 fields in Shropshire in year 1 (November 2015–May 2016), 12 from a wider geographical area in year 2 (August 2016–June 2017; eastern England: Lincolnshire; Central England: Nottinghamshire, Leicestershire; western England: Shropshire, Lancashire), and 5 in year 3 (September 2017–June 2018; Eastern England: Lincolnshire; Central England: Leicestershire; Western England: Shropshire, Lancashire). Re-sampling a sub-set of experimental fields in different years facilitated comparisons between successive crops (Years 1–2 = 4 fields; Years 2–3 = 2 fields; Years 1–3 = 1 field), and the field codes defined in Table 1 identify those in which these sequences occurred. Such resampling resulted in a total of 15 different fields and 22 different crops being studied, the latter comprising 6 different crop types.

### 2.1. Assessment of Slug Distribution

The design of a sampling technique that accurately locates slug patches without prior knowledge of the spatial pattern of population distribution, is problematic. This study used the monitoring approach developed by Petrovskaya et al [31]. In each field, an area of 1 ha was sampled using a 10 × 10 grid of refuge traps (10 m inter-node distance) to assess slug abundance/activity on the soil surface. The minimum distance between the grid and the field boundary was 20 m, and to facilitate its re-establishment in different crops in the rotation, it was mapped using a Global Positioning System (Leica RX1220T, Wetzlar, Germany). The minimum distance from field boundaries reduced the potential for the perimeter to core slug density gradients previously reported in arable fields to differentially affect the accuracy of patch identification across the trapping grid using surface refuge traps [25].

Traps consisted of upturned 18 cm diameter terracotta plant pot saucers (LBS Horticulture Supplies, Lancashire, UK), without bait to avoid slug feeding attraction. In year 1, slug assessments were made at 14-day intervals, reduced thereafter to approximately monthly intervals to accommodate the larger number and more widely dispersed fields sampled in year 2. At each assessment, the number of *D. reticulatum* found under each trap in the sampling grid (i.e., on the soil or the surface of the trap) was recorded, the trap was immediately re-set and any slugs caught were released beneath it.

### 2.2. Spatial and Temporal Stability of Slug Patches within and between Growing Seasons

All statistical analysis was conducted using R Version 4.0.2 [32].

#### 2.2.1. Effect of Field, Year, Growing Region, Crop and Previous Crop on Slug Numbers

Variation between slug counts in different fields within each growing season was investigated using a Poisson mixed effects generalized linear model (GLM).

Annual variability in slug numbers in the 1 ha sampling grids used in this study was investigated using data from field sites in which assessments had been made in multiple cropping seasons (Shrops 2–5, Lancs 1, Leic 1; Table 1). As the datasets did not display a normal distribution, the effect of year on the total number of slugs caught in traps on each assessment date in each field was analyzed using a negative binomial mixed effects GLM.

Following tests for normality, the effects of region, and current and previous crop on the total number of slugs detected during each assessment were also determined using a negative binomial GLM mixed effects model.

#### 2.2.2. Slug Aggregation

The degree of spatial aggregation of slugs was assessed for each growing season (in 2015–16, data was pooled from Shrops 1–5; 2016–17 data pooled from Lancs 1, Leic 1–2, Lincs 1–3, Notts 1, Shrops 2–6; 2017–18 data pooled from Lancs 1, Leic 1, Lincs 4, Shrops 2 and 7; Table 1), and for the pooled data from all three years (2015–18) using Taylor’s Power Law [33]. The log variance and log mean number of slugs per trap were calculated for each assessment date in each of the field sites sampled during the time period. The relationship between log variance and log mean trap counts was then calculated using Pearson’s correlation coefficient, to establish the index of aggregation (the line of best fit).

To investigate the effect of size of trap catches on the index of aggregation when individual assessments in each field were considered, following tests for normality the relationship between the index of aggregation and mean trap count for assessments from all growing seasons was investigated using linear regression.

#### 2.2.3. Hotspot Analysis

Slug distributions within the trapping grid were visualized for each sampling visit to the 22 crops studied using grid maps of counts, generated using the interp and filled.contour functions of R, with the number of slugs in areas between traps estimated by polynomial interpolation. The spatial distribution of slugs caught in the refuge traps within each sampling grid was investigated by identifying statistically significant spatial clusters of higher numbers of slugs using the ScanLRTS function in R. The ‘observed’ distribution (estimated from trap counts) was compared to that ‘expected’ if a homogenous distribution of those slugs was assumed. Areas of significantly higher (*p* < 0.05) than expected slug numbers (hotspots) were highlighted in the visualized grid maps. Data collected during individual assessment visits to each field site were analyzed separately and the number of visits in which significant hotspots occurred were identified and recorded.

#### 2.2.4. Slug Clustering and Patch Stability

To assess the spatial stability of areas of higher slug numbers (patches), following tests for normality, the effect of trap (location within the grid) on slug numbers for each of the 22 individual crops studied was subjected to a Poisson GLM mixed effects model, and the ANOVA function in R was used to extract the main effects. Within each field in which statistically significant effects of trap location on number of slugs caught was found, the correlation between all combinations of sampling grid assessments was then determined using Mantel’s permutation test.

## 3. Results

### 3.1. Effect of Field, Year, Region, Crop and Previous Crop on Slug Numbers

The number of slugs recorded in traps varied between fields within each growing season. In 2015–16, significantly higher slug numbers were recorded at Shrops 4 (equating to 8.33/trap) than in the other fields (0.7–2.88 slugs per trap; F = 12.10, d.f. = 4.41, *p* < 0.001). 

In 2016–17, the mean number of slugs caught in Lincs 3 was significantly lower than Lancs 1 and Shrops 5 (0.07, 0.5 and 1.42 slugs/trap respectively) and significantly higher in Shrops 5 (1.42 slugs per assessment) than Shrops 3, Notts 1, Shrops 4, Shrops 6, Lincs 2 and Shrops 2 (0.21–0.35; F = 3.89, d.f. = 311.77, *p* < 0.001).

In 2017–18, significantly higher trap catches were recorded in Shrops 7 than in the other fields sampled (4.82/trap compared with between 0.27 and 1.00 slugs per assessment (F = 8.14, d.f. = 4.27, *p* < 0.001).

**Figure 1:**
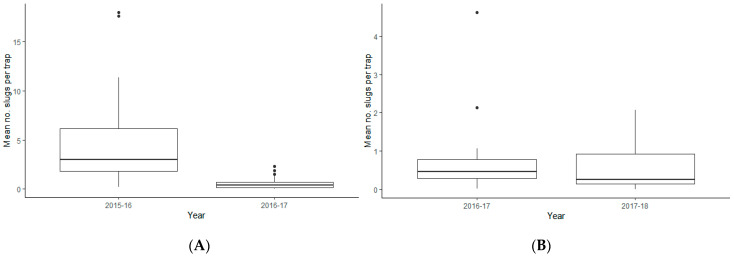
Variation in the number of slugs recorded (mean No. trap^−1^) in successive seasons in a 10 × 10 grid of refuge traps, set within a rectangular 1ha plot in each field. (**A**) Fields assessed in both 2015–16 and 2016–17 (Shrops 2, Shrops 3, Shrops 4 and Shrops 5); (**B**) Fields assessed in both 2016–17 and 2017–18 (Shrops 2, Leic 1, Lancs 1). For details of crops sampled see Table 1. Horizontal line = median x slugs per trap; bottom and top of box indicate the 25th and 75th percentiles; whiskers = 1.5*x* the interquartile range above or below the 25th or 75th percentiles; points are outliers.

Where assessments were carried out in the same fields in different growing seasons significantly higher slug activity was recorded in 2015–16 than in the 2016–17 seasons (Χ^2^ = 81.66, d.f. = 1, *p* < 0.001; Figure 1A) but no significant difference was found between trap catches in the 2016–17 and 2017–18 seasons (Χ^2^ = 0.84, d.f. = 1, *p* = 0.36; Figure 1B). 

**Figure 2:**
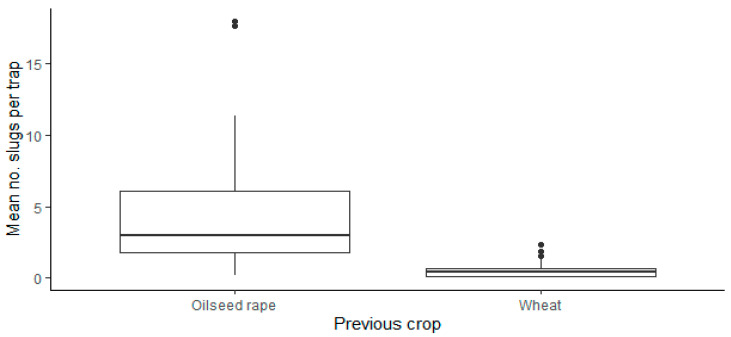
Variation in the total number of slugs recorded in fields where the previous crop was either oilseed rape (11 Fields) or cereals (9 Fields). Slug activity reported as the mean No. slugs per trap in a 10 × 10 grid of refuge traps, set within a rectangular 1ha plot in each field/crop; for details of crops sampled see Table 1. Horizontal line = median x slugs per trap; bottom and top of box indicate the 25th and 75th percentiles; whiskers = 1.5*x* the interquartile range above or below the 25th or 75th percentiles; points are outliers.

There was no significant effect of region (Χ^2^ = 3.14, d.f. = 2.80, *p* = 0.21)) or crop (Χ^2^ = 6.00, d.f.= 3.80, *p* = 0.11) on the number of slugs recorded in the traps; however, there was a significant effect of the previous crop (Χ^2^ = 13.87, d.f. = 1.80, *p* < 0.001) with numbers higher in those fields where the preceding crop was oilseed rape than when winter wheat had been grown (Figure 2).

### 3.2. Slug Aggregation

**Figure 3:**
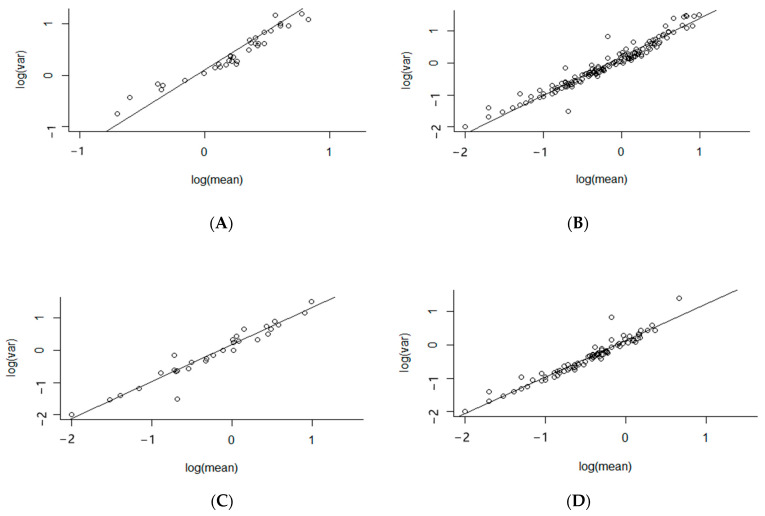
The relationship between log (variance) and log (mean) number of slugs per trap recorded in the 10 × 10 grid of refuge traps, set within a rectangular 1ha plot in each field assessed in (**A**) 2015–16, (**B**) 2016–17, (**C**) 2017–18 and (**D**) all three years. Each point on the graphs represents one assessment date in one field.

The highest aggregation index across a growing season was calculated using data from 2015–16, the year in which the highest mean slug counts were recorded, with a lower index recorded in the two years with lower populations (Table 2, Figure 3). If a species has a regular distribution the index of aggregation tends towards zero [33], with an index exceeding 1 reflecting progressively greater aggregation.

When data from individual fields was investigated, the mean trap count had a significant effect on the index of aggregation (t = 5.3, d.f. = 1,19, *p* < 0.001), with a higher index observed on individual assessment dates with higher slug counts. Thus, the patchy distribution of slugs may be more readily identified using refuge traps when there is higher slug activity on the soil surface.

### 3.3. Hotspot Analysis

Following confirmation of the aggregated distribution of slugs within the 1 ha plots established in study fields, the location of discrete domains with significantly (*p* < 0.05) higher numbers of slugs than would be expected if a random distribution is assumed, were identified using hotspot analysis and visualized using grid maps (Table 3; Figure 4; Figures S1–S22).

Higher density domains were detected in either the majority, or all of the assessment visits to each of the 22 individual field crops investigated over the three years of this study (Table 3). Of the total of 167 assessment visits to these sites, higher density domains were recorded in 162 visits, with no such domains identified in only 5 assessments. These five visits coincided with periods of very low slug activity on the soil surface as recorded by refuge traps (Maximum = a mean of 0.07 slugs per trap). The lack of an effect of geographical location of the field on spatial distribution of slugs was evident, with significant heterogenous distributions recorded in 98.4% of the 129 sampling visits made to fields in the Western half of England, and 92.1% (of 38) in the dryer East.

### 3.4. Spatial Stability of Patches

In 11 of the 22 crops studied, consistent differences between the catches of individual traps were detected, indicating potential spatial clustering of slug activity, and reflecting the higher density domains identified by hotspot analysis. These included all five fields assessed in 2015/16, 3 of the 12 sites in 2016/17, and 3 of the five sites in 2017/18 (Table 4). A characteristic common to 10 of the 11 fields in which a more homogenous distribution of slug catches was recorded was a low slug infestation in crops (mean <1.5 slugs per trap). This was reflected in a catch of >4 slugs (the current AHDB treatment threshold in winter wheat and oilseed rape in the UK) not being recorded in any individual trap in these fields. The remaining field was located on land that was still in the recovery phase following reclamation from a former opencast mining site with associated significant soil disturbance.

The grids of trapping nodes used in this study generated a data matrix describing the pattern of spatial clustering of slugs that are active on the soil surface on each assessment date. To investigate the degree of similarity between the location of the resultant slug patches defined by the data matrices collected on different assessment dates at the same site, the relationship between the numerical separations for all the possible pairs of trap samples taken on different dates was established using Mantel’s permutation test [34]. The degree of correlation between the location of the slug patches in pairs of matrices from different sampling dates is a measure of spatial stability of the patches across the assessment period.

Comparisons were made of all permutations of pairs of data matrices at each of the 11 study sites in which GLM had detected consistent (statistically significant) effects of trap location on number of slugs caught. The results indicate that a significant correlation between the pairs of matrices occurred in only 35.7% of the comparisons (Table 5(A); Tables S1–S11). Taking account of the level of surface activity of slugs in the crops studied, however, in 68.4% of the permutations in which a significant correlation between the pairs of matrices was not found, one or both of those matrices had a mean trap catch of ≤1.5 slugs/trap. This figure rose to 92.9% where mean trap catch was lower than the current UK treatment threshold of 4/trap (Table 5(B)). Thus, when lower numbers of slugs are active on the soil surface, differences between the catches of surface refuge traps may be insufficient to detect the effect of trap location on number of slugs caught. When higher numbers of slugs were surface active, however, consistent effects of trap location on catches were readily detected. Hence, patches displayed spatio-temporal stability throughout the growing season of the crop, although they could not always be detected using surface refuge traps, for example when adverse weather conditions resulted in a large proportion of the slug population being below the soil surface.

## 4. Discussion

The heterogenous distribution of slugs in arable fields may offer the potential for reducing molluscicide use while maintaining effective control of slug damage in commercial crops by spatially targeting applications to specific areas containing higher slug numbers [2,3,24,29]. If this potential is to be realized, such slug patches should (i) be a characteristic of populations in all crops that are susceptible to damage, (ii) display sufficient spatio-temporal stability, and (iii) be identifiable using a cost-effective method. Current evidence demonstrating that slug patches are often found in arable crops and are stable (at least within a growing season) is based on data collected from relatively few crops and usually over short time periods, and requires empirical confirmation. In the current work the existence of identifiable slug patches was investigated using hotspot analysis to locate areas with significantly higher than expected slug numbers than would be predicted if a random distribution was assumed. Higher density patches were identified in all of the 22 commercial crops (six crop types) studied, and in all but six (3.6%) of the assessment visits undertaken. Failure to locate any patches occurred in just a single visit to each of six different fields, and in each case slug counts were very low at the time of the assessment (<0.07 slugs per trap). No effect of region on the spatial distribution of slugs within fields was evident when western and eastern parts of the UK were compared. Thus, this study critically re-examines previous findings [2,3,24,25] that slugs display a heterogeneous distribution in arable fields, to prove that the existence of patches of higher slug numbers separated by areas with lower slug activity is a characteristic property occurring across a range of crop types and geographical regions. Increased slug damage has been shown to follow significant slug surface activity [26], and this was confirmed in higher density slug patches in the current study [24]. After seed germination, however, rapid production of new leaves makes the use of metrics such as percentage leaf damage unreliable as a method of identifying patch location and edges.

The intermittent failure to locate slug patches in sequential assessments of the same field has also been noted in a previous study [24] and requires explanation. Several methods for assessing slug populations have been developed with soil washing, soil flooding, defined area traps and refuge trapping being most commonly used for research [16,25,35,36,37]. Although their efficacy for establishing absolute slug populations is limited, there is evidence that surface refuge traps can be used successfully to determine the relative numbers of slugs that are active on the soil surface in different areas of fields, and were thus selected for use in trapping grids in the current work [25]. Slug activity on the soil surface (thus catches) are known to be affected by environmental factors such as soil moisture and temperature, which vary throughout the growing season [33,38,39,40]. For example, periods with temperatures of <13 or >17 °C [41], or prolonged periods of low rainfall can result in a significant proportion of the population retreating to a protected environment below the soil surface where they cannot be detected using refuge traps until more favourable conditions return [25]. Thus, the failure to detect any patches in six of the assessments may have been the result of adverse physical conditions that resulted in the observed very low trap catches and associated small differences between the number of slugs recorded in individual traps in the grid. Further, successful suppression of slug populations following application of slug pellets to some fields may have contributed to masking of the location of the patches prone to harboring higher slug numbers by significantly lowering trap catches across the sampling grid (particularly within those patches). The fields used in this study were all situated on commercial farms, and all had received molluscicide treatments. Thus although, this may in a few cases have affected detection of slug patches, the results of the hot spot analysis suggest that treatments did not prevent their formation and cohesion.

In the current study, mean trap catches were higher in 2015/16 than in either 2016/17 or 2017/18, the years in which generally lower temperatures were noted at field sites. No significant relationships between slug numbers and growing region or the crop assessed were recorded but a significant effect of previous crop was found with higher numbers caught following oilseed rape, confirming previous reports [42]. The latter finding should be treated with some caution, however, as five of the eleven crops that followed oilseed rape were sampled in 2015/16, with no other rotation represented in this year. The highest slug numbers of any of the three years were recorded in 2015/16, but the possibility of other confounding factors operating in this year cannot be ignored. The index of aggregation calculated from Taylor’s Power Law was also higher in 2015/16 than in the other two field seasons, mirroring the difference in slug activity in the crops sampled. This may also be the result of the use of refuge traps making patches of higher slug numbers more difficult to detect due to smaller numerical differences between catches of traps within and between patches when activity on the soil surface was lower. The contention that low population size resulted in greater difficulty in identifying patches by grid trapping with refuge traps was supported by the analysis of within-season stability of slug distributions. Consistent differences between the catches at individual nodes of trapping grids were found to occur on multiple assessment dates in 11 of the 22 crops studied. These included all five crops assessed in the year with higher slug numbers (2015/16), and three crops in each of the other two growing seasons, and suggest spatial clustering of slug activity. Further, 10 of the 11 fields with lower correlations between distribution of slug catches were characterized by having low slug infestations (<1.5 slugs per trap), with the final crop being established on soil that had been subjected to significant disturbance.

A more detailed analysis compared the pattern of spatial clustering described by the data matrices of slug numbers recorded on each assessment date in each of the 11 crops in which consistent differences had been identified. Significant correlations between the cluster (patch) locations were readily located in fields with higher slug numbers. In more than 90% of the cases where significant correlations were not obtained, mean trap catches in one or both data matrices were lower than 4/trap, the current UK treatment threshold in winter wheat and oilseed rape. Thus, where accurate data on slug surface activity was available, patches displayed spatio-temporal stability throughout the period in which crop assessments were made, although they could not be detected using surface refuge traps during periods of adverse weather conditions when a large proportion of the slug population would have been situated below the soil surface. Under such circumstances, however, the results of this study suggest that the difficulty of detection would not occur when slug numbers exceed current action thresholds, so it would be unlikely to adversely affect the concept of targeted treatments.

In the context of commercial agriculture, perimeter-core slug density gradients that can result in a broad zone of generally higher slug numbers near crop edges, may allow easier detection of patches using refuge traps in such areas [25]. If, however, environmental factors influence the locations in which patches form [24], such gradients are unlikely to significantly affect their number or position. In practice, most molluscicide applications are made during a period encompassing the establishment and early growth of crops, but information regarding the timing of the formation of slug aggregations is sparse. Thus, work focused on the spatial stability of slug patches during the period spanning two sequential crops in commercial rotations is now required.

The mobility of slugs is key to understanding the mechanisms underpinning the formation and cohesion of population patches and thus their temporal stability. A recent study in which movement of individual slugs was followed for a period of five weeks using radio frequency identification technology, demonstrated that most (~80%) foraged within a limited area, with the mean distance travelled from the release point being 78.7 ± 33.7 cm in spring and 101.9 ± 24.1 cm in autumn [27]. This suggests that the limited locomotor behaviour may promote the patchy distribution observed during the crop growing season in the current work. Few studies have addressed the behavioural mechanisms leading to the limited foraging area of the gray field slug but, in other organisms, density dependent individual movement is known to contribute to pronounced spatial heterogeneity and the stability of patches containing higher population density [43,44,45,46,47]. Preliminary work on the gray field slug has indicated that, within areas of higher slug density (i.e., in the presence of conspecifics), individuals move more slowly and for shorter distances, spend more time immobile, and display a strongly biased distribution of turning angles. It was shown that, consistently with these factors, the area occupied by a patch grows more slowly in the presence of higher slug densities, arguably indicating that density dependence of individual slug movement enhances patch stability [28]. In summary of the three pre-requisites for effective targeting of molluscicides at patches of higher slug densities in crops, which are defined at the start of this section, two have been met. Slug patches were (i) detected in all assessments in which activity was sufficiently high to enable differentiation of refuge trap catches, and despite difficulties related to intermittent detection between assessments (linked to periods of adverse environmental conditions), their locations were (ii) stable within crop growing seasons. However, pre-requisite (iii) remains a challenge. The efficacy of refuge traps varies with the environmental conditions prior to assessments and although they remain suitable for use in the current threshold-based approach to sustainable molluscicide use in the UK [29,48] the associated risk of individual assessments missing some patches make them commercially unsuitable for accurate targeting of pesticide applications at areas of a field containing higher slug densities. The behavioural mechanisms reinforcing patch cohesion are beginning to be understood, and ongoing work is investigating the effect of edaphic heterogeneity as a driver of patch location, which may provide a basis for targeted application of molluscicides.

## 5. Conclusions

The gray field slug, *D. reticulatum*, is known to display a heterogenous distribution in arable crops, characterized by discrete patches of higher slug densities dispersed across the field, with fewer slugs in intervening areas. In recent years, there has been increasing interest in the potential for reducing pesticide use on farms by spatially targeting molluscicide applications for pest management only at these patches. 

If this potential is to be realized, then the slug patches should (i) be a characteristic of populations in all crops that are susceptible to damage, (ii) display sufficient spatio-temporal stability, and (iii) a cost-effective method of locating the patches is required.

This study tested the hypotheses that (a) slug patches can be identified in a range of commonly grown arable crops in major crop growing regions of the UK, and (b) the location of higher density patches within fields remains stable throughout the crop growing season.

Slug patches were detected by hot spot analysis in all the 22 field crops (6 crop types) studied, and in 162 of the 167 individual assessment visits to those crops. Detection of patches using the surface refuge traps selected for the study became more difficult during periods of lower slug activity. 

Despite difficulties related to intermittent detection of patches between assessment dates, which was linked with low slug activity on the soil surface (probably due to periods of adverse environmental conditions), their locations were shown to be stable within crop growing seasons. 

Thus, two of the three requirements defined above have been confirmed. To address the third criterium, other work investigating the use of combinations of physical characteristics of soil that may be linked to patch location is ongoing.

## Figures and Tables

**Figure 4:**
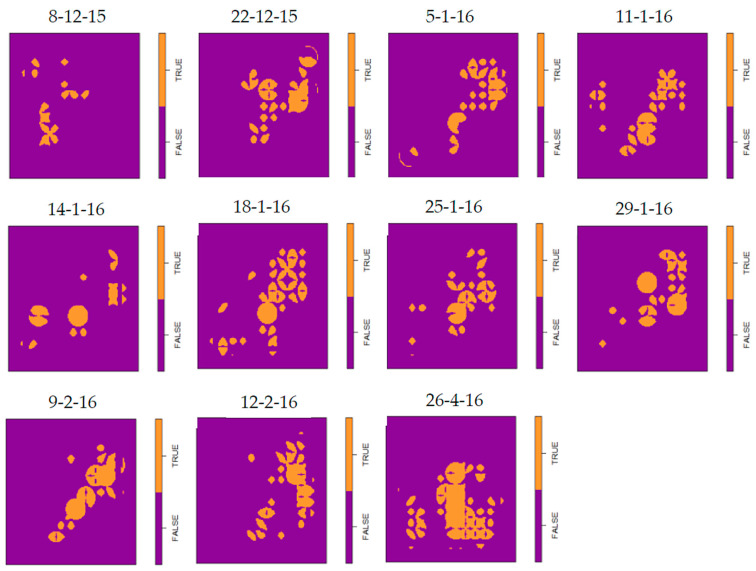
Visualized grid map showing the location of domains of higher slug density identified by hotspot analysis of data collected from a 10 × 10 grid of refuge traps, set within a rectangular 1ha plot at the Shrops 2 field site between December 2015 and April 2016. Data for each of the 11 assessments made at this site were analyzed separately; grid squares colored in amber indicate the location of discrete domains with significantly (*p* < 0.05) higher numbers of slugs than would be expected if a random distribution is assumed. Dates above each map indicate the day on which the assessment was made. For grid maps from all field sites used in the study see Figures S1–S22. The Shrops 2 field site was sampled in each of three consecutive growing cycles and for comparison equivalent grid maps for crops grown in the 2016/17 and 2017/18 cropping seasons are provided in Figures S6 and S18.

**Table 1:** Location, field codes and crop rotations of experimental field sites in each year of the study. Crops in bold were used in experiments; crops in italics were grown prior to the commencement of experimental work.

County (Field Code)	Location	Cropping Season			
	(Nearest Town/Village)	2014–15	2015–16	2016–17	2017–18
Shropshire (Shrops 1)	Adeney (1)	*Oilseed rape*	**Winter wheat**		
Shropshire (Shrops 2)	Adeney (2)	*Oilseed rape*	**Winter wheat**	**Winter barley**	**Oilseed rape**
Shropshire (Shrops 3)	Lynn (1)	*Oilseed rape*	**Winter wheat**	**Fallow**	
Shropshire (Shrops 4)	Lynn (2)	*Oilseed rape*	**Winter wheat**	**Fallow**	
Shropshire (Shrops 5)	Uppington (1)	*Oilseed rape*	**Winter wheat**	**Fallow**	
Leicestershire (Leic 1)	Oadby		*Oilseed rape*	**Winter wheat**	**Cover crop** **(black oat/phacelia)**
Leicestershire (Leic 2)	Hoby		*Oilseed rape*	**Winter wheat**	
Lancashire (Lancs 1)	Wigan		*Oilseed rape*	**Winter wheat**	**Fallow**
Lincolnshire (Lincs 1)	South Kyme (1)		*Oilseed rape*	**Winter wheat**	
Lincolnshire (Lincs 2)	South Kyme (2)		*Spring wheat*	**Spring wheat**	
Lincolnshire (Lincs 3)	Dog Dyke		*Winter wheat*	**Winter wheat**	
Nottinghamshire (Notts 1)	Flawborough		*Oilseed rape*	**Winter wheat**	
Shropshire (Shrops 6)	Bridgnorth		*Winter wheat*	**Oilseed rape**	
Shropshire (Shrops 7)	Uppington (2)			*Oilseed rape*	**Winter wheat**
Lincolnshire (Lincs 4)	Belchford			*Spring beans*	**Winter wheat**

**Table 2:** The Taylor’s Power Law Index of Aggregation for individual years of the study, and for all three years combined. Mean slug catches of refuge traps (No. per trap), regression equation and *R*^2^ values for the relationship between log (variance) vs. log (mean) calculated from x trap count and variance for each assessment date in each field.

Year	Mean Slug Count (No./Trap)	Regression	*R* ^2^	*N*	Index of Aggregation
2015–16	3.93	*y* = 0.11*x* + 1.08 (see also Figure 3A)	0.92	46	1.52
2016–17	0.56	*y* = 0.10*x* + 1.00 (see also Figure 3B)	0.94	87	1.09
2017–18	1.57	*y* = 0.06*x* + 1.10 (see also Figure 3C)	0.92	30	1.15
2015–18	1.68	*y* = 0.10*x* + 1.12 (see also Figure 3D)	0.94	163	1.21

**Table 3:** The number of assessment visits to each field in which hot spot analysis identified discrete domains containing significantly higher slug density (*p* < 0.05; HD) when compared with that expected if the population was randomly distributed across the sampling grid. Assessments made using a 10 by 10 grid of refuge traps set within a rectangular 1ha plot.

County/Field	Season	HD Domain	No HD Domain	County/Field	Season	HD Domain	No HD Domain
**Shropshire**				**Leicestershire**			
Shrops 1	2015–16	7	0	Leic 1	2016–17	8	0
Shrops 2	2015–16	11	0	Leic 1	2017–18	5	0
Shrops 2	2016–17	8	1	Leic 2	2016–17	7	0
Shrops 2	2017–18	8	0	**Lancashire**			
Shrops 3	2015–16	7	0	Lancs 1	2016–17	10	0
Shrops 3	2016–17	5	0	Lancs 1	2017–18	4	1
Shrops 4	2015–16	13	0	**Lincolnshire**			
Shrops 4	2016–17	5	0	Lincs 1	2016–17	8	1
Shrops 5	2015–16	8	0	Lincs 2	2016–17	8	0
Shrops 5	2016–17	7	0	Lincs 3	2016–17	7	1
Shrops 6	2016–17	7	0	Lincs 4	2017–18	5	1
Shrops 7	2017–18	7	0	**Nottinghamshire**			
				Notts 1	2016–17	7	0

**Table 4:** Spatial clustering of slug activity identified by catches of surface refuge traps arranged in a 10 × 10 grid of refuge traps, set within a rectangular 1ha plot in each field, and mean number of slugs caught, in 22 field sites from three growing seasons. The effect of trap location within the grid on slug numbers was investigated using a Poisson GLM mixed effects model, with main effects extracted using the Anova function in R (Χ^2^_;_ *p*). Consistent differences between the catches of individual traps (indicating potential spatial clustering of slug activity) are highlighted in bold, and mean trap catches exceeding an arbitrary “low population size” of 1.5 per trap in bold/italics.

Site	Years	Χ^2^	*p*	Mean Trap Count
Shrops 1	2015–16	136.50	***p* < 0.01**	0.7
Shrops 2	2015–16	82.31	***p* < 0.001**	***6.8***
Shrops 3	2015–16	136.16	***p* < 0.001**	***3.4***
Shrops 4	2015–16	14.69	***p* < 0.001**	***11.4***
Shrops 5	2015–16	18.20	***p* < 0.001**	***3.7***
Lancs 1	2016–17	157.70	***p* < 0.001**	***2.1***
Shrops 2	2016–17	11.47	***p* < 0.001**	0.1
Shrops 3	2016–17	0.05	*p* > 0.05	0.1
Shrops 4	2016–17	0.30	*p* > 0.05	0.2
Shrops 5	2016–17	20.99	***p* < 0.001**	***1.9***
Shrops 6	2016–17	1.35	*p* > 0.05	0.4
Leic 1	2016–17	0.01	*p* >0.05	0.6
Leic 2	2016–17	0.09	*p* > 0.05	1.2
Lincs 1	2016–17	1.88	*p* > 0.05	0.5
Lincs 2	2016–17	2.08	*p* > 0.05	0.2
Lincs 3	2016–17	0.92	*p* > 0.05	0.1
Notts 1	2016–17	0.02	*p* > 0.05	0.05
Lancs 1	2017–18	0.01	*p* > 0.05	***2.1***
Shrops 2	2017–18	14.01	***p* < 0.001**	0.1
Shrops 7	2017–18	6.03	***p* < 0.05**	***3.4***
Leic 1	2017–18	2.24	*p* > 0.05	0.3
Lincs 4	2017–18	6.79	***p* < 0.01**	**1.0**

**Table 5:** Temporal stability of higher density slug patches at the 11 study sites in which a GLM mixed effects model detected consistent effects of trap location on number of slugs caught between data matrices generated by assessments taken on different dates. Tables show (**A**) the percentage of permutations of pairs of data matrices at each study site in which a significant correlation between the pairs of matrices was recorded and (**B**) the percentage of permutations in which a significant correlation between the pairs of matrices was not recorded but one or both assessments were taken when slug surface activity resulted in mean catches of <4 slugs per trap.

**(A) the percentage of permutations of pairs of data matrices at each study site in which a significant correlation between the pairs of matrices was recorded**								
**Year**	**Lancs 1**	**Shrops 1**	**Shrops 2**	**Shrops 3**	**Shrops 4**	**Shrops 5**	**Shrops 7**	**Lincs 4**
2015–16	-	19.0	61.8	28.6	65.4	14.3	-	-
2016–17	22.2	-	10.7	-	-	33.3	-	-
2017–18	-	-	14.3	-	-	-	13.3	0.0
**(B) the percentage of permutations in which a significant correlation between the pairs of matrices was not recorded but one or both assessments were taken when slug surface activity resulted in mean catches of <4 slugs per trap**								
**Year**	**Lancs 1**	**Shrops 1**	**Shrops 2**	**Shrops 3**	**Shrops 4**	**Shrops 5**	**Shrops 7**	**Lincs 4**
2015–16	-	100.0	100.0	100.0	40.7	100.0	-	-
2016–17	100.0	-	100.0	-	-	100.0	-	-
2017–18	-	-	100.0	-	-	-	100.0	100.0

## Data Availability

The datasets used and/or analyzed during the current study are available from the corresponding author on reasonable request.

## Supplementary Materials

The following are available online at https://www.mdpi.com/2075-4450/12/1/9/s1, Figures S1–S22: Visualized grid maps showing locations of domains of higher slug density identified by hotspot analysis of data collected from rectangular grids of 100 refuge traps established at the 22 field sites used in this study, Tables S1–S11: Results of Mantel’s Permutation tests investigating spatio-temporal stability of areas of higher slug density within a regular 10 × 10 m (1 ha) sampling grid established in the 11 field sites investigated in this study.
