# Supplementary material for: Stability of Patches of Higher Population Density within the Heterogenous Distribution of the Gray Field Slug Deroceras reticulatum in Arable Fields in the UK
Source: Insects. 2020 Dec 25;12(1):9. doi: 10.3390/insects12010009 (PMC7823367; doi:10.3390/insects12010009)
Supplement: Supplementary file 1 [file insects-12-00009-s001.zip › supplementary-xml/Supplementary Figure 1.pdf]

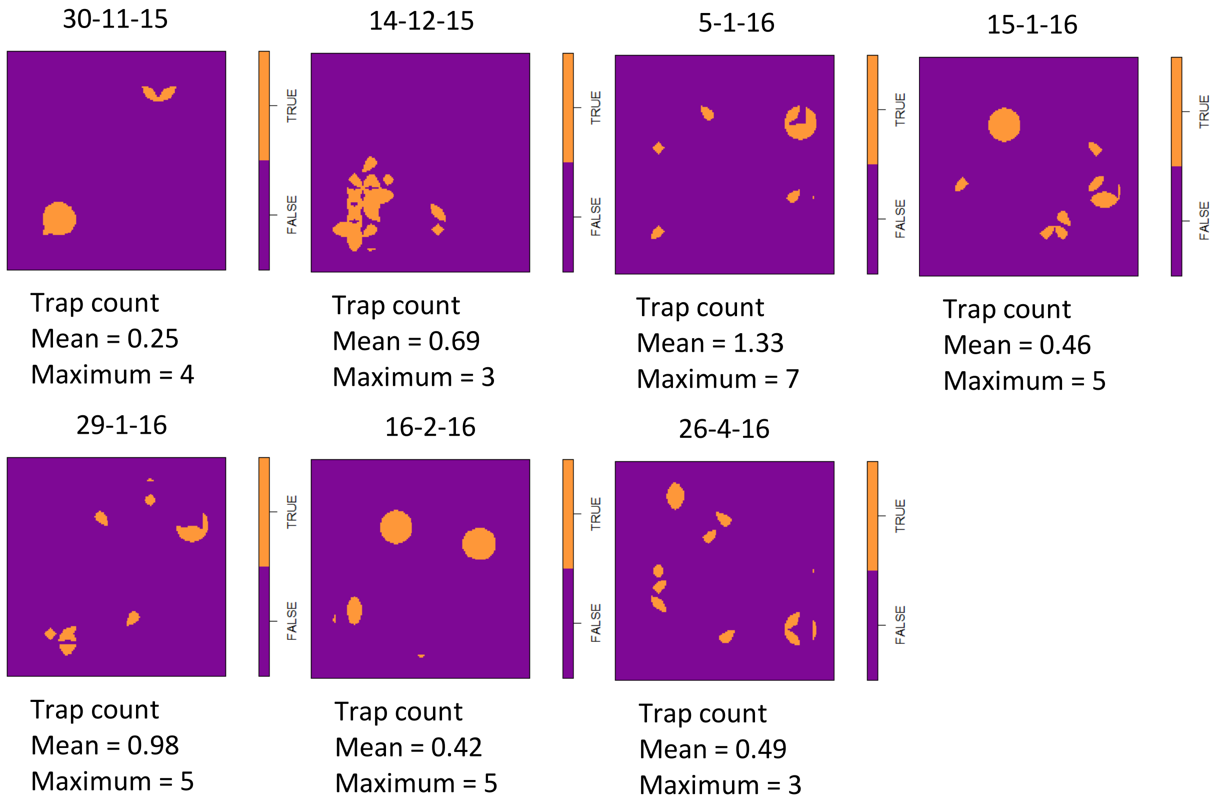

Figure S1. Visualized grid maps showing the location of domains of higher slug density identified by hotspot analysis of data collected from the rectangular grid of 100 refuge traps established at the Shrops 1 field site during the 2015-16 field season. Data for each of the 7 assessments made at this site were analysed separately; grid squares coloured in amber indicate the location of discrete domains with significantly ($P<0.05$) higher numbers of slugs than would be expected if a random distribution is assumed. Mean trap count = mean number of slugs caught in the 100 traps in the sampling grid; Maximum trap count = largest number of slugs caught by an individual trap.

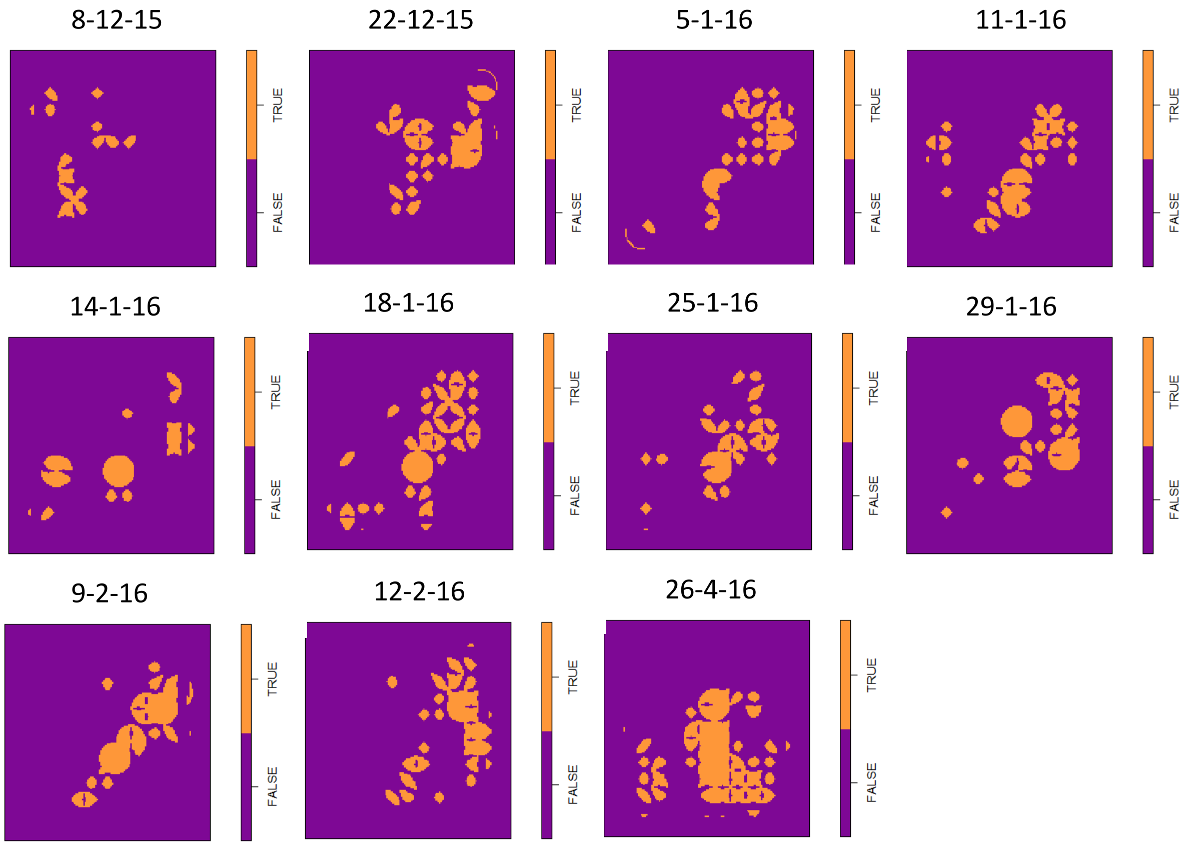

Figure S2. Visualized grid maps showing the location of domains of higher slug density identified by hotspot analysis of data collected from the rectangular grid of 100 refuge traps established at the Shrops 2 field site during the 2015-16 field season. Data for each of the 11 assessments made at this site were analysed separately; grid squares coloured in amber indicate the location of discrete domains with significantly ($P<0.05$) higher numbers of slugs than would be expected if a random distribution is assumed. Mean trap count = mean number of slugs caught in the 100 traps in the sampling grid; Maximum trap count = largest number of slugs caught by an individual trap.

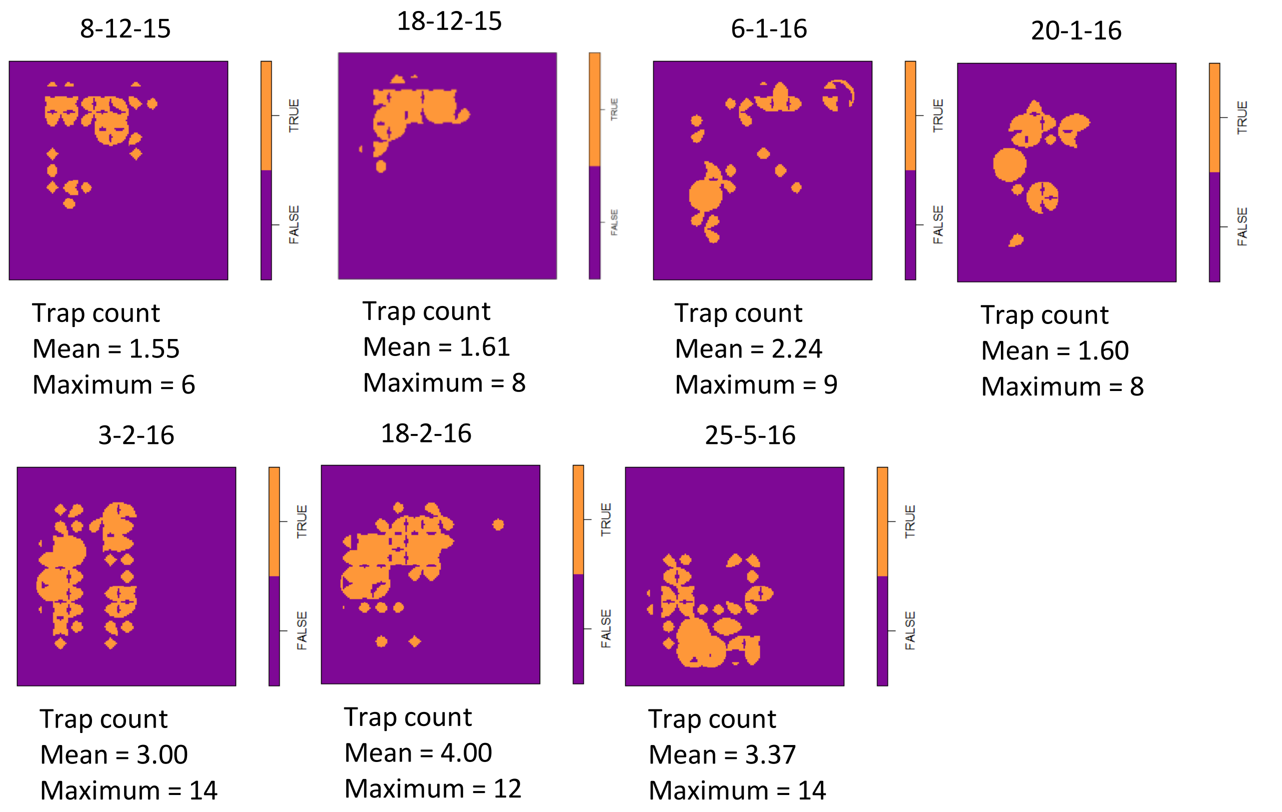

Figure S3. Visualized grid maps showing the location of domains of higher slug density identified by hotspot analysis of data collected from the rectangular grid of 100 refuge traps established at the Shrops 3 field site during the 2015-16 field season. Data for each of the 7 assessments made at this site were analysed separately; grid squares coloured in amber indicate the location of discrete domains with significantly ($P<0.05$) higher numbers of slugs than would be expected if a random distribution is assumed. Mean trap count = mean number of slugs caught in the 100 traps in the sampling grid; Maximum trap count = largest number of slugs caught by an individual trap.

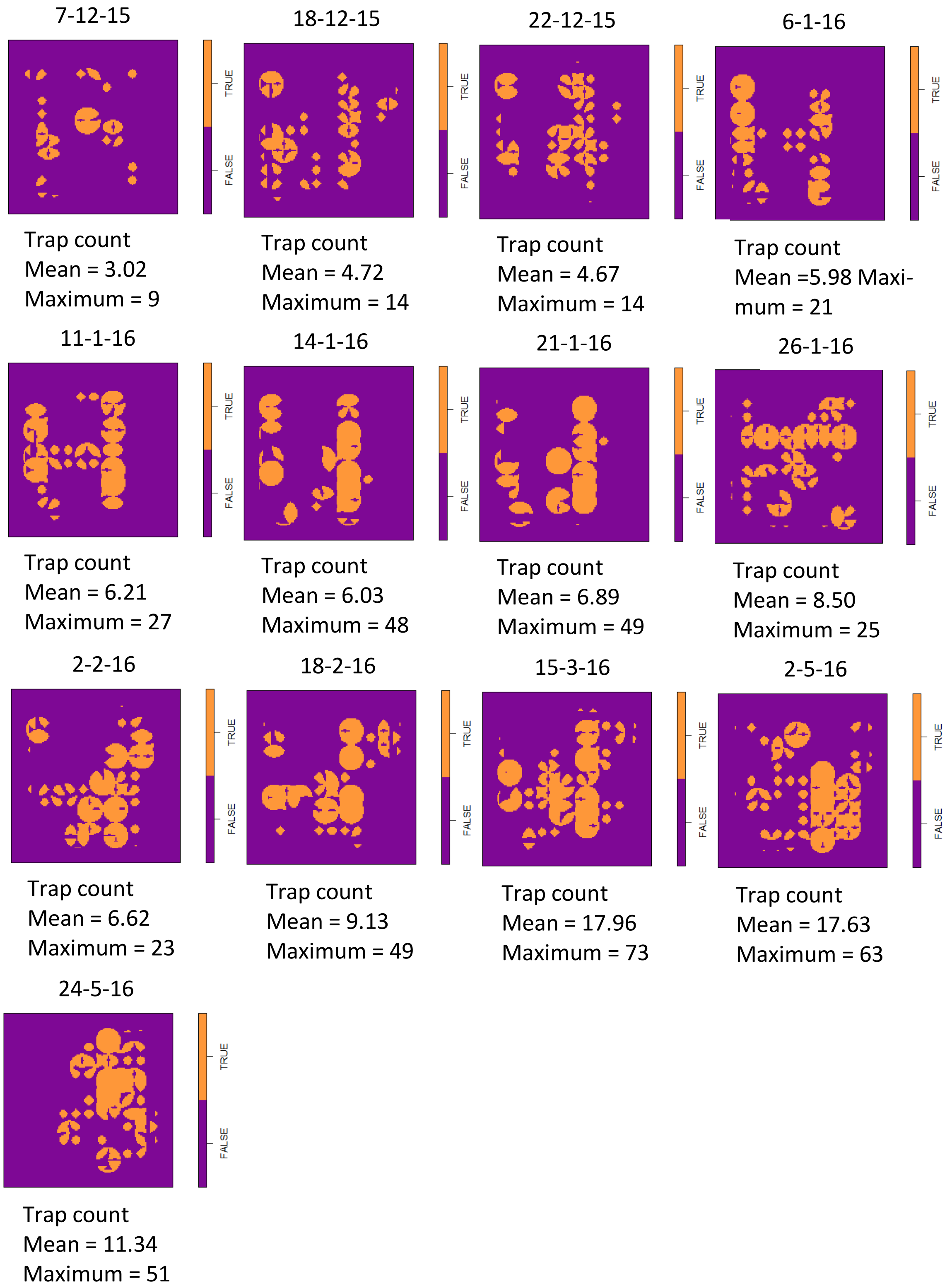

Figure S4. Visualized grid maps showing the location of domains of higher slug density identified by hotspot analysis of data collected from the rectangular grid of 100 refuge traps established at the Shrops 4 field site during the 2015-16 field season. Data for each of the 13 assessments made at this site were analysed separately; grid squares coloured in amber indicate the location of discrete domains with significantly (P<0.05) higher numbers of slugs than would be expected if a random distribution is assumed. Mean trap count = mean number of slugs caught in the 100 traps in the sampling grid; Maximum trap count = largest number of slugs caught by an individual trap.

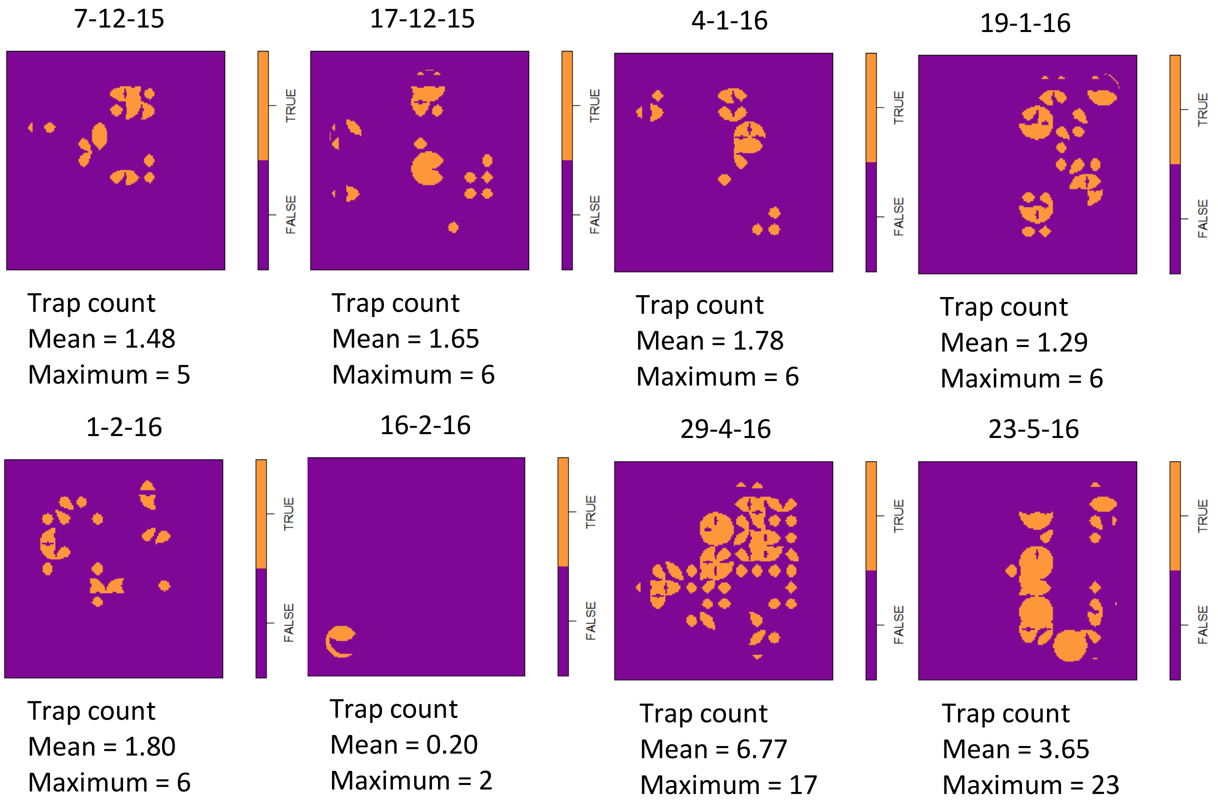

Figure S5. Visualized grid maps showing the location of domains of higher slug density identified by hotspot analysis of data collected from the rectangular grid of 100 refuge traps established at the Shrops 5 field site during the 2015-16 field season. Data for each of the 8 assessments made at this site were analysed separately; grid squares coloured in amber indicate the location of discrete domains with significantly (P<0.05) higher numbers of slugs than would be expected if a random distribution is assumed. Mean trap count = mean number of slugs caught in the 100 traps in the sampling grid; Maximum trap count = largest number of slugs caught by an individual trap.

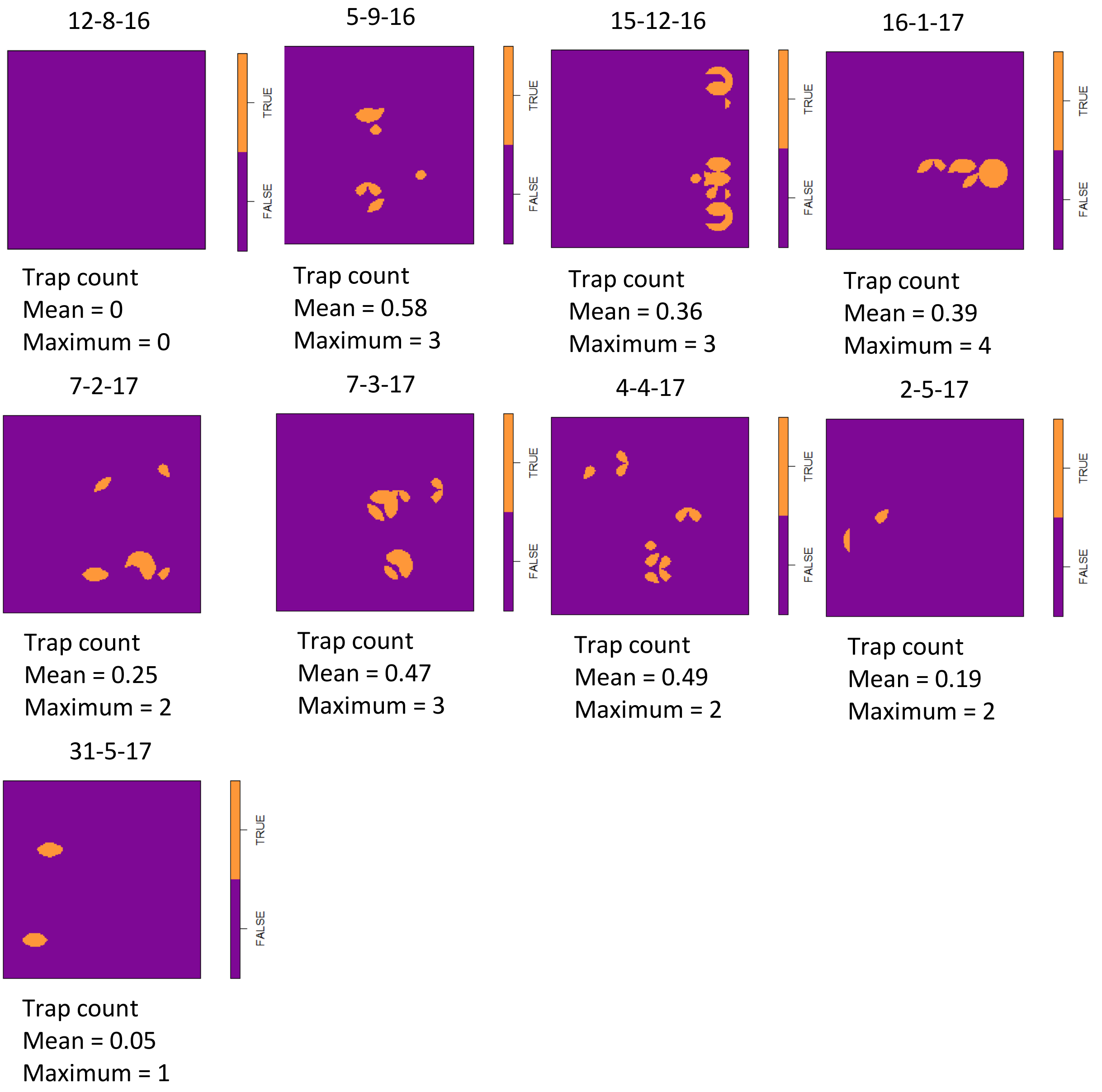

Figure S6. Visualized grid maps showing the location of domains of higher slug density identified by hotspot analysis of data collected from the rectangular grid of 100 refuge traps established at the Shrops 2 field site during the 2016-17 field season. Data for each of the 9 assessments made at this site were analysed separately; grid squares coloured in amber indicate the location of discrete domains with significantly ($P<0.05$) higher numbers of slugs than would be expected if a random distribution is assumed. Mean trap count = mean number of slugs caught in the 100 traps in the sampling grid; Maximum trap count = largest number of slugs caught by an individual trap.

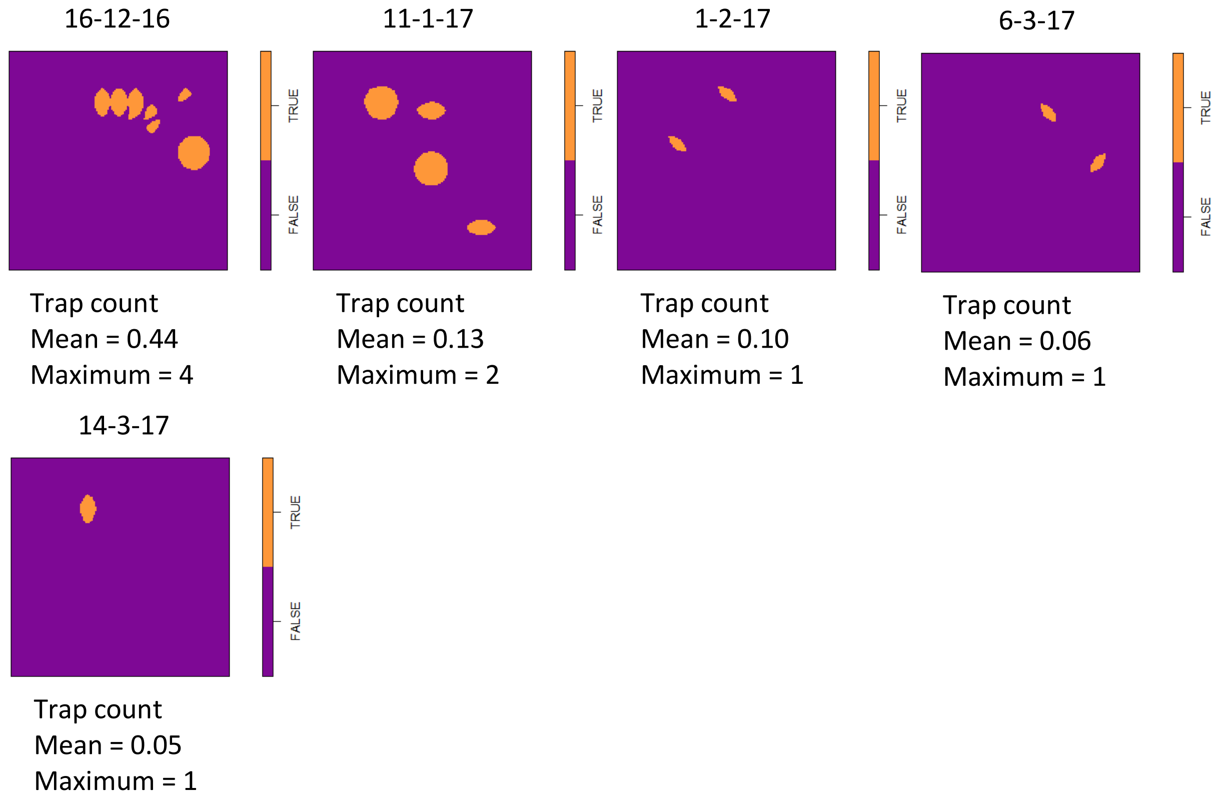

Figure S7. Visualized grid maps showing the location of domains of higher slug density identified by hotspot analysis of data collected from the rectangular grid of 100 refuge traps established at the Shrops 3 field site during the 2016-17 field season. Data for each of the 5 assessments made at this site were analysed separately; grid squares coloured in amber indicate the location of discrete domains with significantly ($P<0.05$) higher numbers of slugs than would be expected if a random distribution is assumed. Mean trap count = mean number of slugs caught in the 100 traps in the sampling grid; Maximum trap count = largest number of slugs caught by an individual trap.

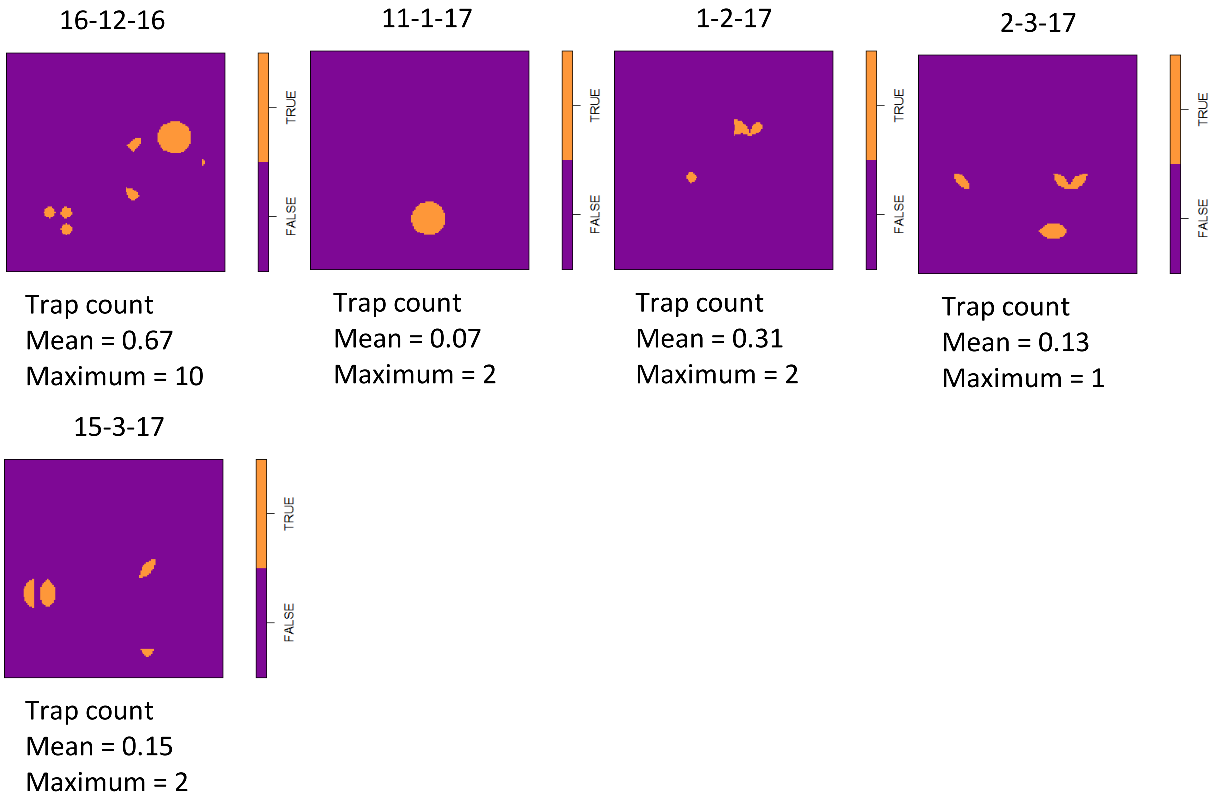

Figure S8. Visualized grid maps showing the location of domains of higher slug density identified by hotspot analysis of data collected from the rectangular grid of 100 refuge traps established at the Shrops 4 field site during the 2016-17 field season. Data for each of the 5 assessments made at this site were analysed separately; grid squares coloured in amber indicate the location of discrete domains with significantly ($P<0.05$) higher numbers of slugs than would be expected if a random distribution is assumed. Mean trap count = mean number of slugs caught in the 100 traps in the sampling grid; Maximum trap count = largest number of slugs caught by an individual trap.

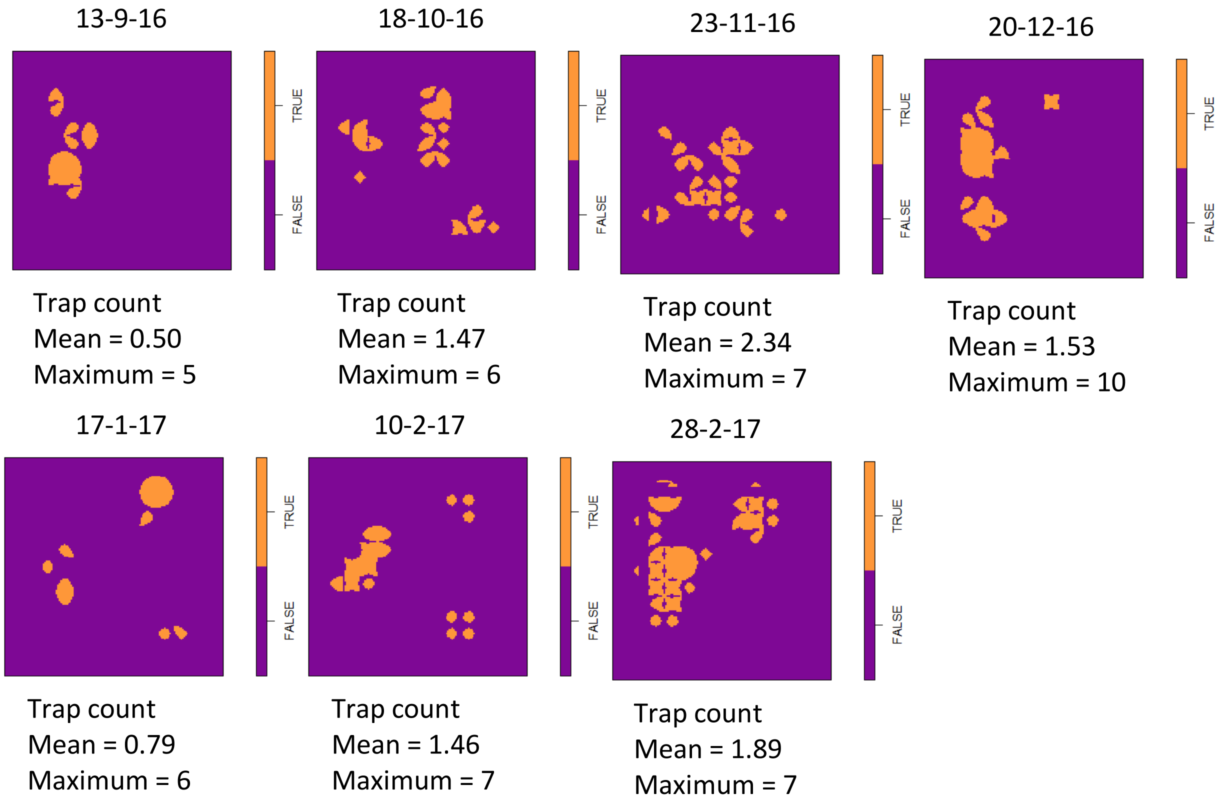

Figure S9. Visualized grid maps showing the location of domains of higher slug density identified by hotspot analysis of data collected from the rectangular grid of 100 refuge traps established at the Shrops 5 field site during the 2016-17 field season. Data for each of the 7 assessments made at this site were analysed separately; grid squares coloured in amber indicate the location of discrete domains with significantly ($P<0.05$) higher numbers of slugs than would be expected if a random distribution is assumed. Mean trap count = mean number of slugs caught in the 100 traps in the sampling grid; Maximum trap count = largest number of slugs caught by an individual trap.

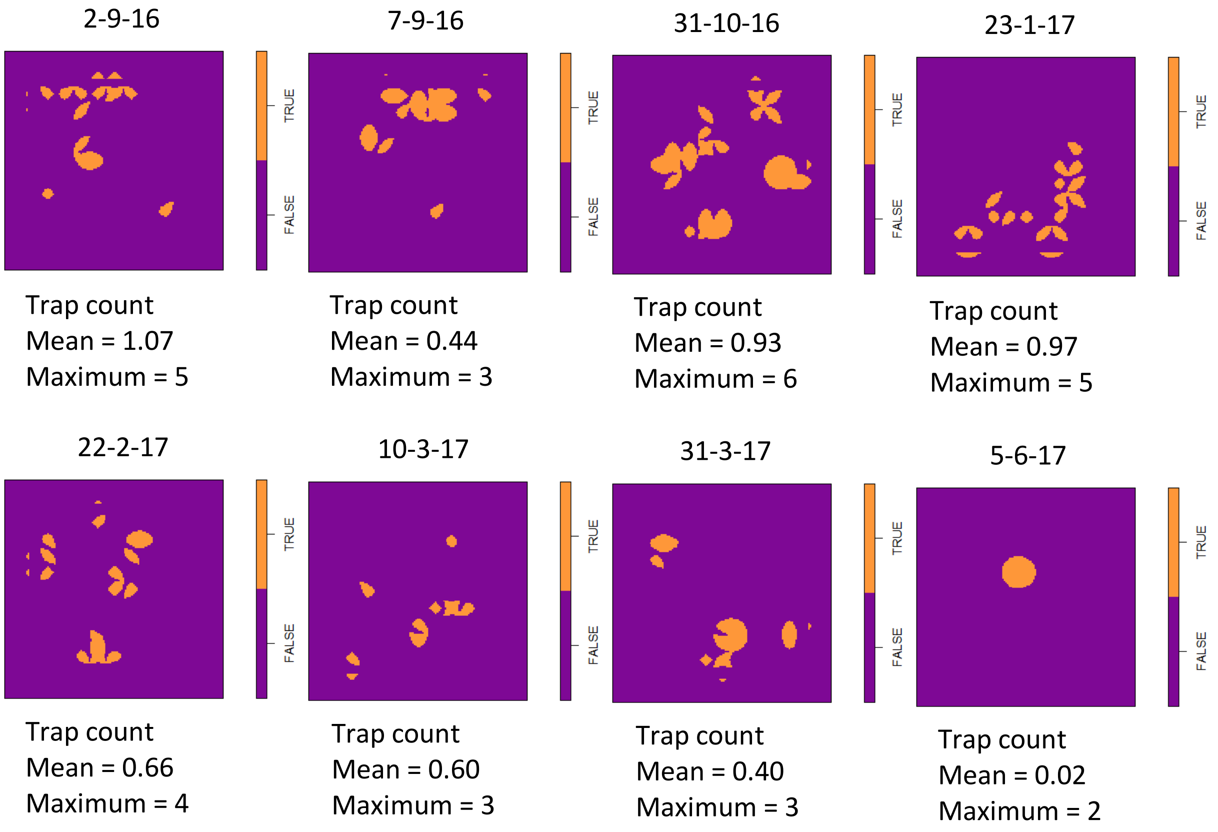

Figure S10. Visualized grid maps showing the location of domains of higher slug density identified by hotspot analysis of data collected from the rectangular grid of 100 refuge traps established at the Leic 1 field site during the 2016-17 field season. Data for each of the 8 assessments made at this site were analysed separately; grid squares coloured in amber indicate the location of discrete domains with significantly ($P<0.05$) higher numbers of slugs than would be expected if a random distribution is assumed. Mean trap count = mean number of slugs caught in the 100 traps in the sampling grid; Maximum trap count = largest number of slugs caught by an individual trap.

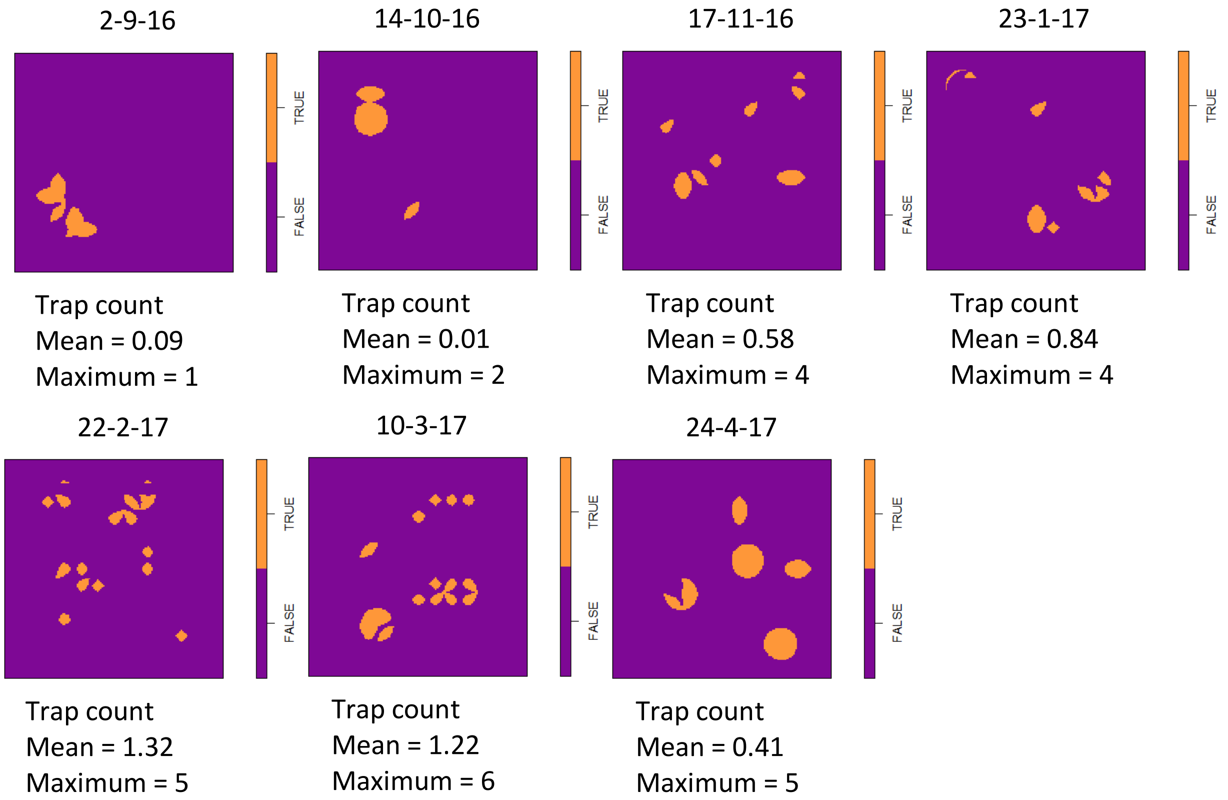

Figure S11. Visualized grid maps showing the location of domains of higher slug density identified by hotspot analysis of data collected from the rectangular grid of 100 refuge traps established at the Leic 2 field site during the 2016-17 field season. Data for each of the 7 assessments made at this site were analysed separately; grid squares coloured in amber indicate the location of discrete domains with significantly ($P<0.05$) higher numbers of slugs than would be expected if a random distribution is assumed. Mean trap count = mean number of slugs caught in the 100 traps in the sampling grid; Maximum trap count = largest number of slugs caught by an individual trap.

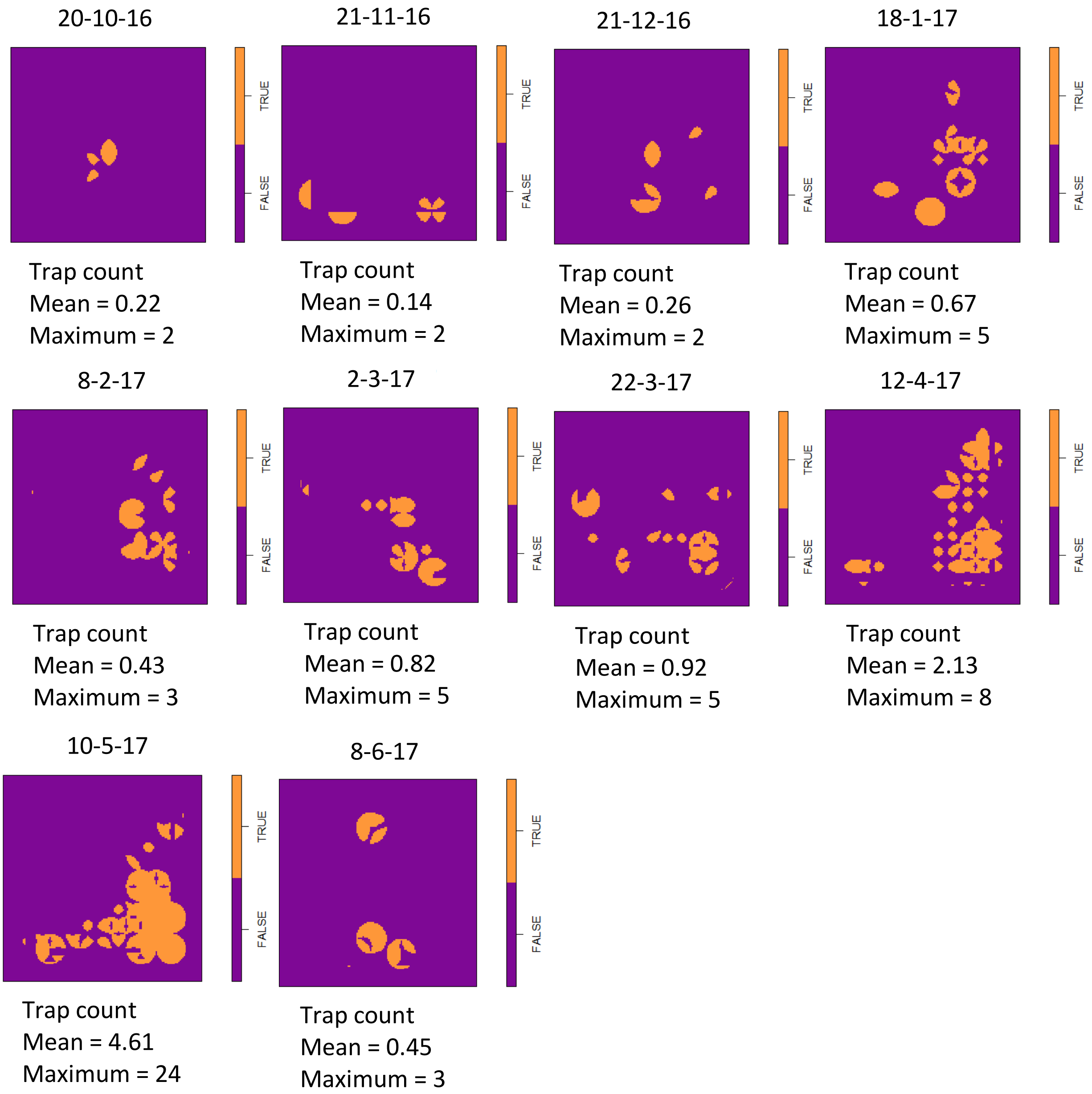

Figure S12. Visualized grid maps showing the location of domains of higher slug density identified by hotspot analysis of data collected from the rectangular grid of 100 refuge traps established at the Lancs 1 field site during the 2016-17 field season. Data for each of the 10 assessments made at this site were analysed separately; grid squares coloured in amber indicate the location of discrete domains with significantly ($P<0.05$) higher numbers of slugs than would be expected if a random distribution is assumed. Mean trap count = mean number of slugs caught in the 100 traps in the sampling grid; Maximum trap count = largest number of slugs caught by an individual trap.

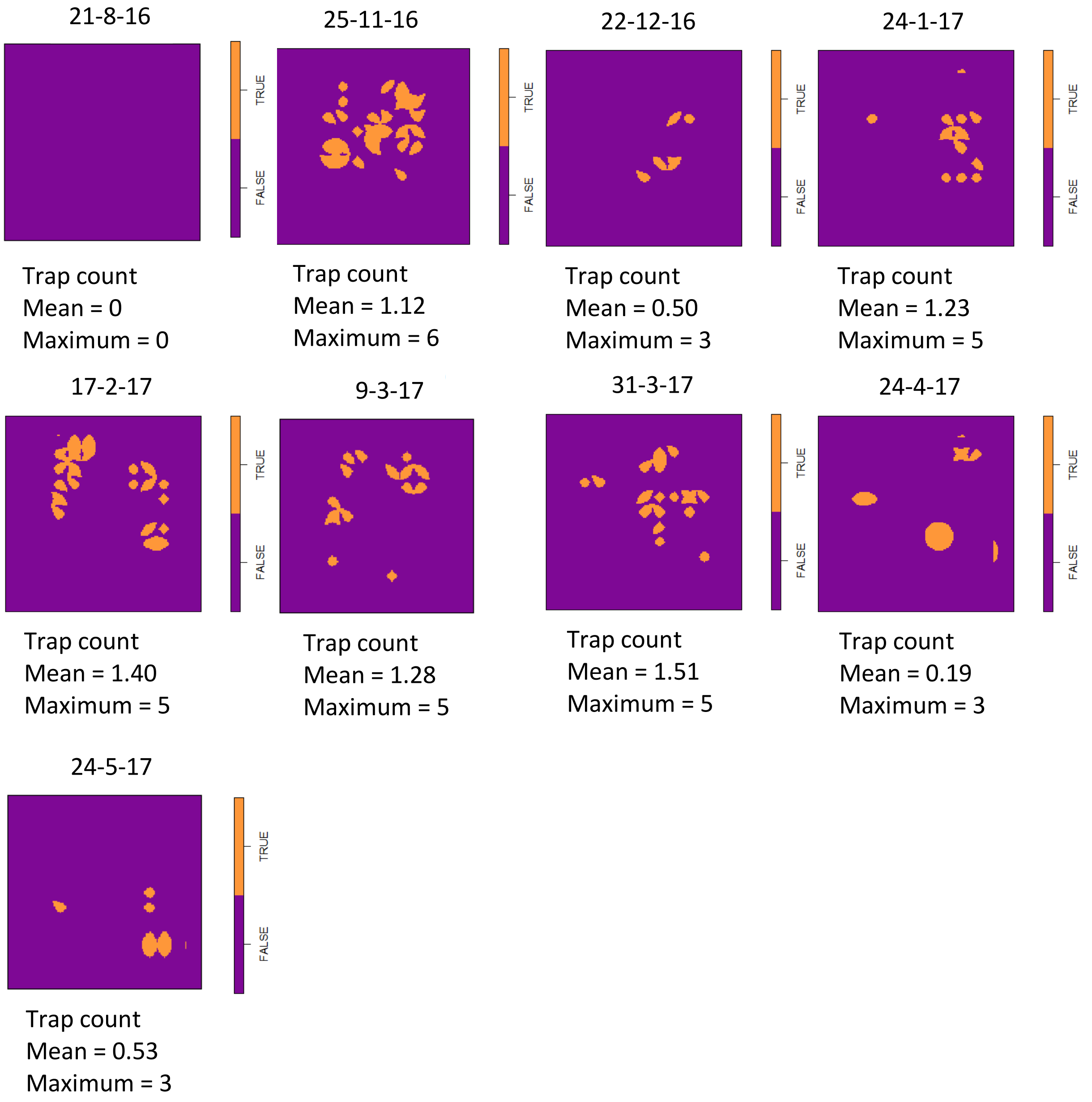

Figure S13. Visualized grid maps showing the location of domains of higher slug density identified by hotspot analysis of data collected from the rectangular grid of 100 refuge traps established at the Lincs 1 field site during the 2016-17 field season. Data for each of the 9 assessments made at this site were analysed separately; grid squares coloured in amber indicate the location of discrete domains with significantly ($P<0.05$) higher numbers of slugs than would be expected if a random distribution is assumed. Mean trap count = mean number of slugs caught in the 100 traps in the sampling grid; Maximum trap count = largest number of slugs caught by an individual trap.

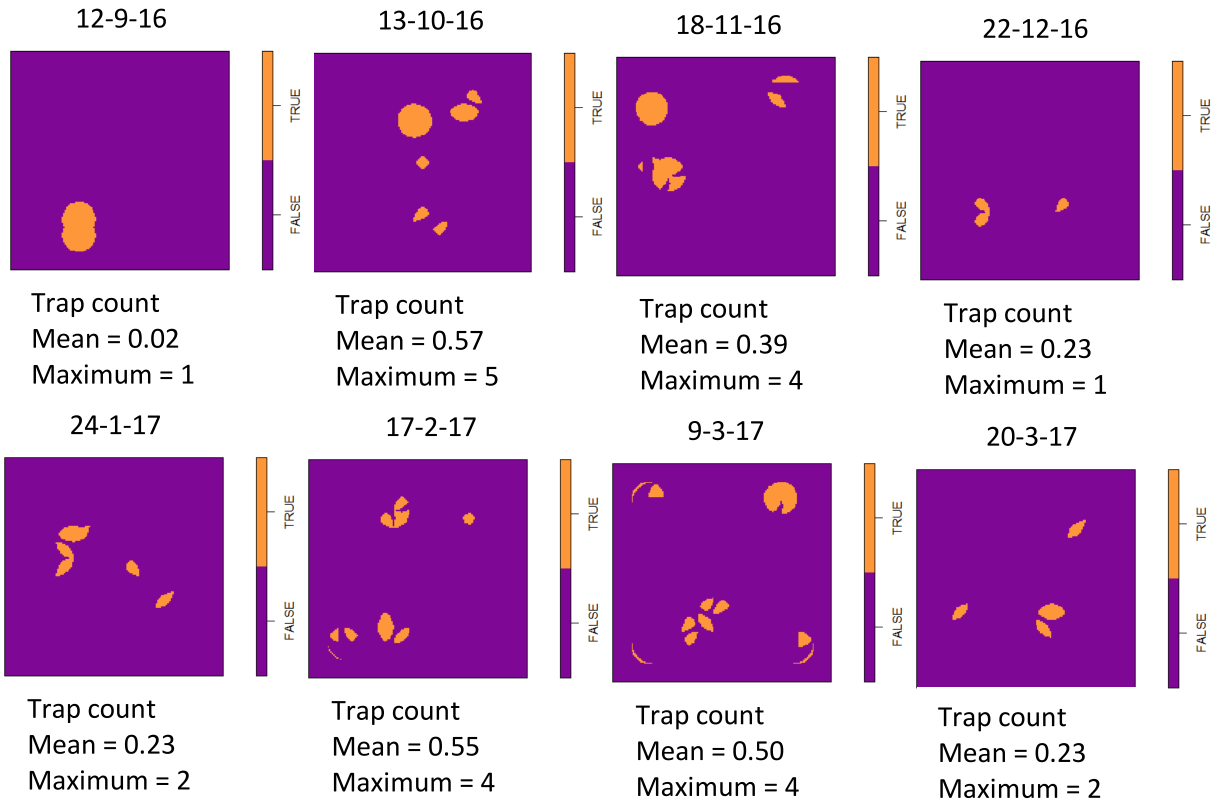

Figure S14. Visualized grid maps showing the location of domains of higher slug density identified by hotspot analysis of data collected from the rectangular grid of 100 refuge traps established at the Lincs 2 field site during the 2016-17 field season. Data for each of the 8 assessments made at this site were analysed separately; grid squares coloured in amber indicate the location of discrete domains with significantly ($P<0.05$) higher numbers of slugs than would be expected if a random distribution is assumed. Mean trap count = mean number of slugs caught in the 100 traps in the sampling grid; Maximum trap count = largest number of slugs caught by an individual trap.

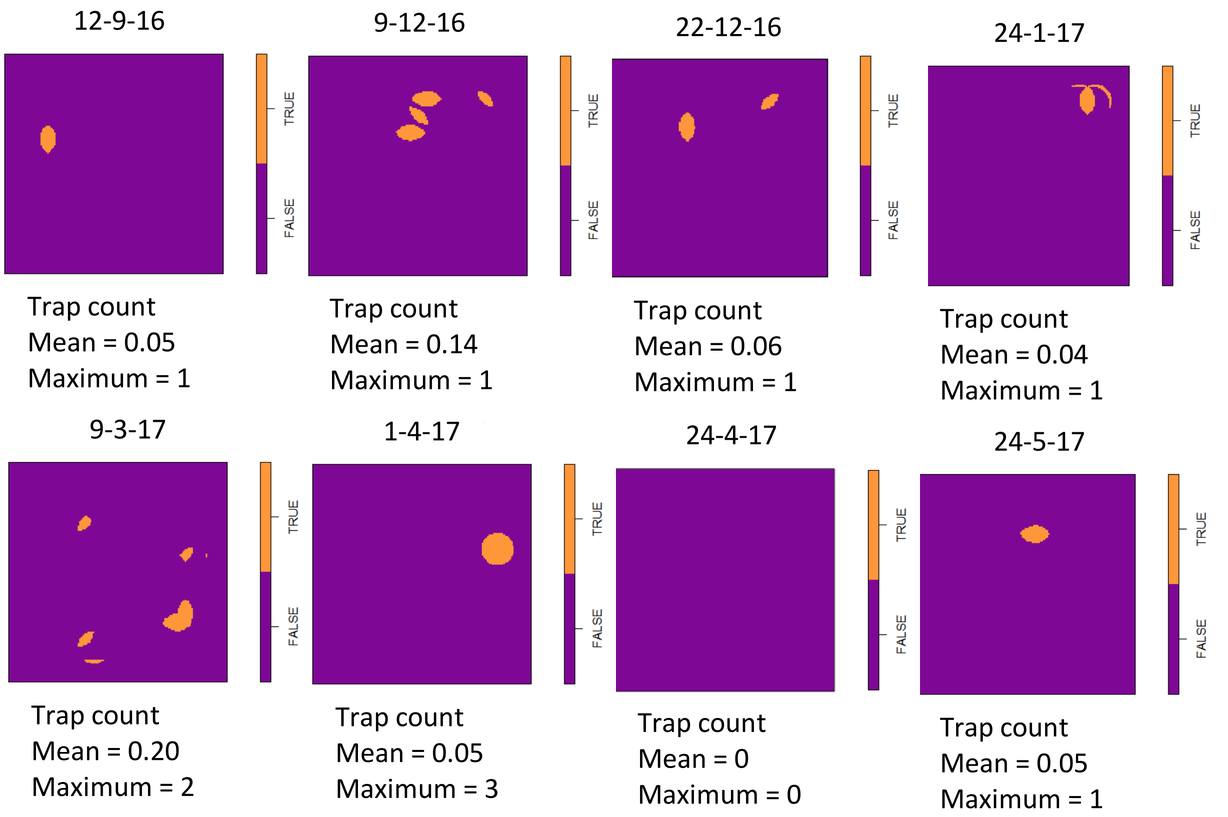

Figure S15. Visualized grid maps showing the location of domains of higher slug density identified by hotspot analysis of data collected from the rectangular grid of 100 refuge traps established at the Lincs 3 field site during the 2016-17 field season. Data for each of the 8 assessments made at this site were analysed separately; grid squares coloured in amber indicate the location of discrete domains with significantly ($P<0.05$) higher numbers of slugs than would be expected if a random distribution is assumed. Mean trap count = mean number of slugs caught in the 100 traps in the sampling grid; Maximum trap count = largest number of slugs caught by an individual trap.

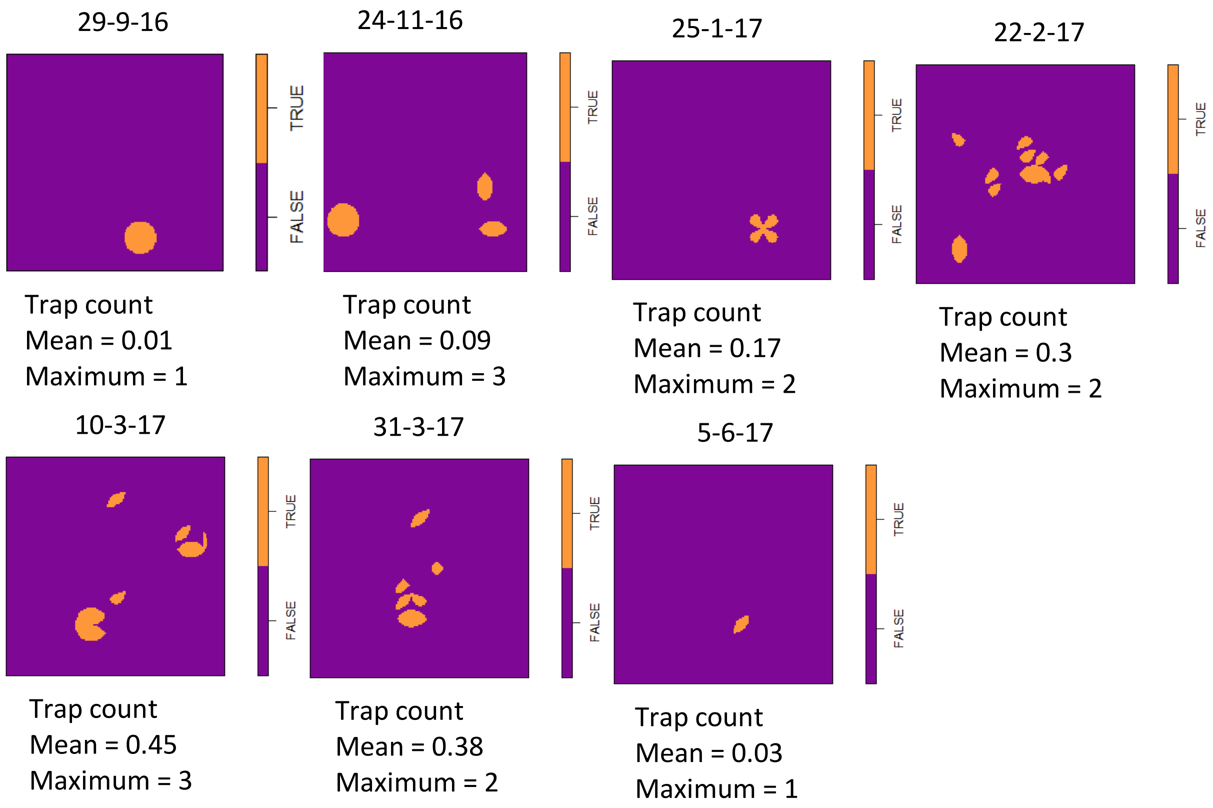

Figure S16. Visualized grid maps showing the location of domains of higher slug density identified by hotspot analysis of data collected from the rectangular grid of 100 refuge traps established at the Notts 1 field site during the 2016-17 field season. Data for each of the 7 assessments made at this site were analysed separately; grid squares coloured in amber indicate the location of discrete domains with significantly ($P<0.05$) higher numbers of slugs than would be expected if a random distribution is assumed. Mean trap count = mean number of slugs caught in the 100 traps in the sampling grid; Maximum trap count = largest number of slugs caught by an individual trap.

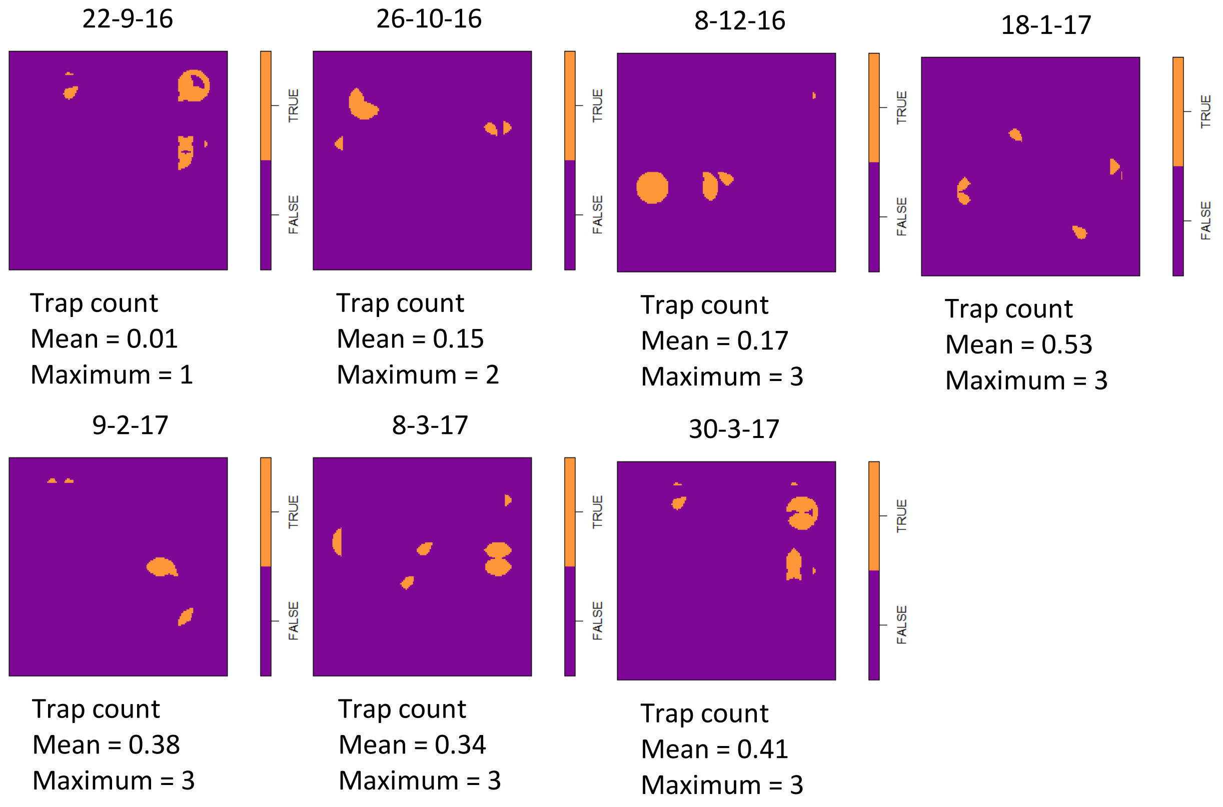

Figure S17. Visualized grid maps showing the location of domains of higher slug density identified by hotspot analysis of data collected from the rectangular grid of 100 refuge traps established at the Shrops 6 field site during the 2016-17 field season. Data for each of the 7 assessments made at this site were analysed separately; grid squares coloured in amber indicate the location of discrete domains with significantly ($P<0.05$) higher numbers of slugs than would be expected if a random distribution is assumed. Mean trap count = mean number of slugs caught in the 100 traps in the sampling grid; Maximum trap count = largest number of slugs caught by an individual trap.

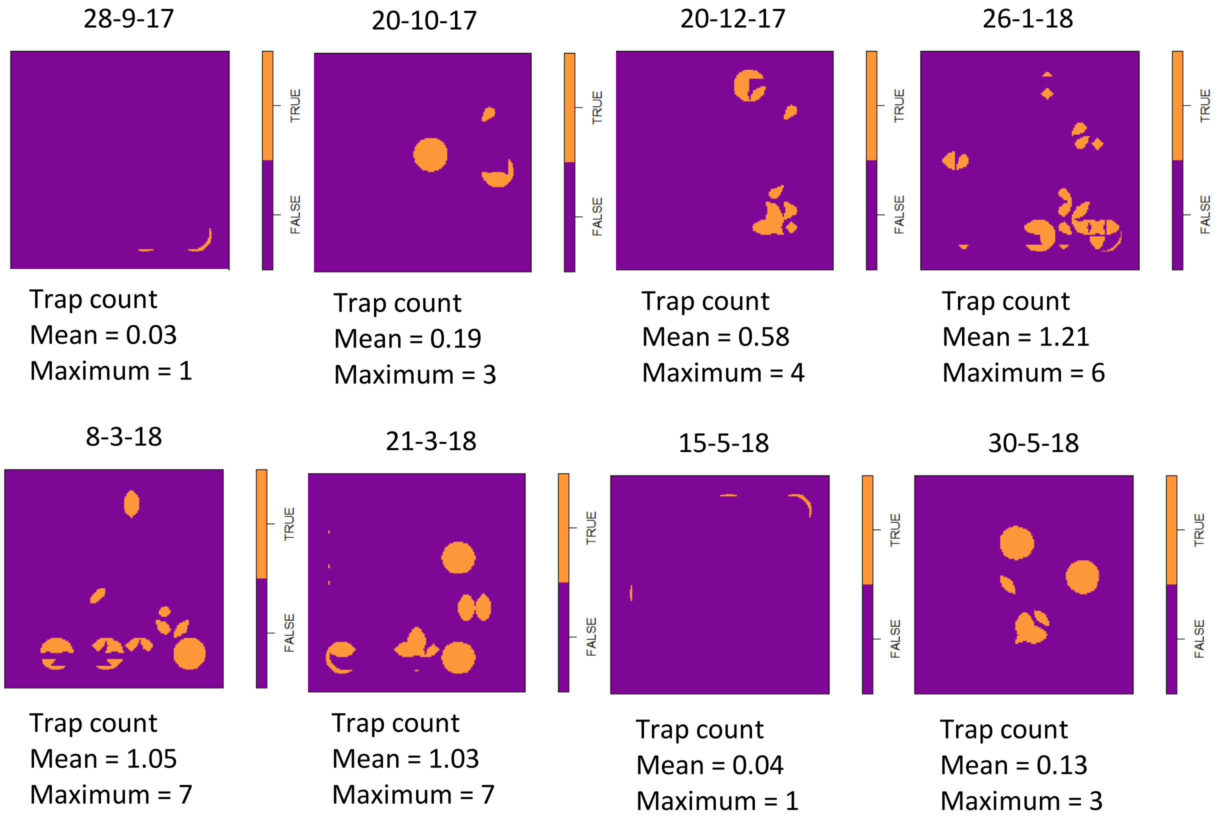

Figure S18. Visualized grid maps showing the location of domains of higher slug density identified by hotspot analysis of data collected from the rectangular grid of 100 refuge traps established at the Shrops 2 field site during the 2017-18 field season. Data for each of the 8 assessments made at this site were analysed separately; grid squares coloured in amber indicate the location of discrete domains with significantly ($P<0.05$) higher numbers of slugs than would be expected if a random distribution is assumed. Mean trap count = mean number of slugs caught in the 100 traps in the sampling grid; Maximum trap count = largest number of slugs caught by an individual trap.

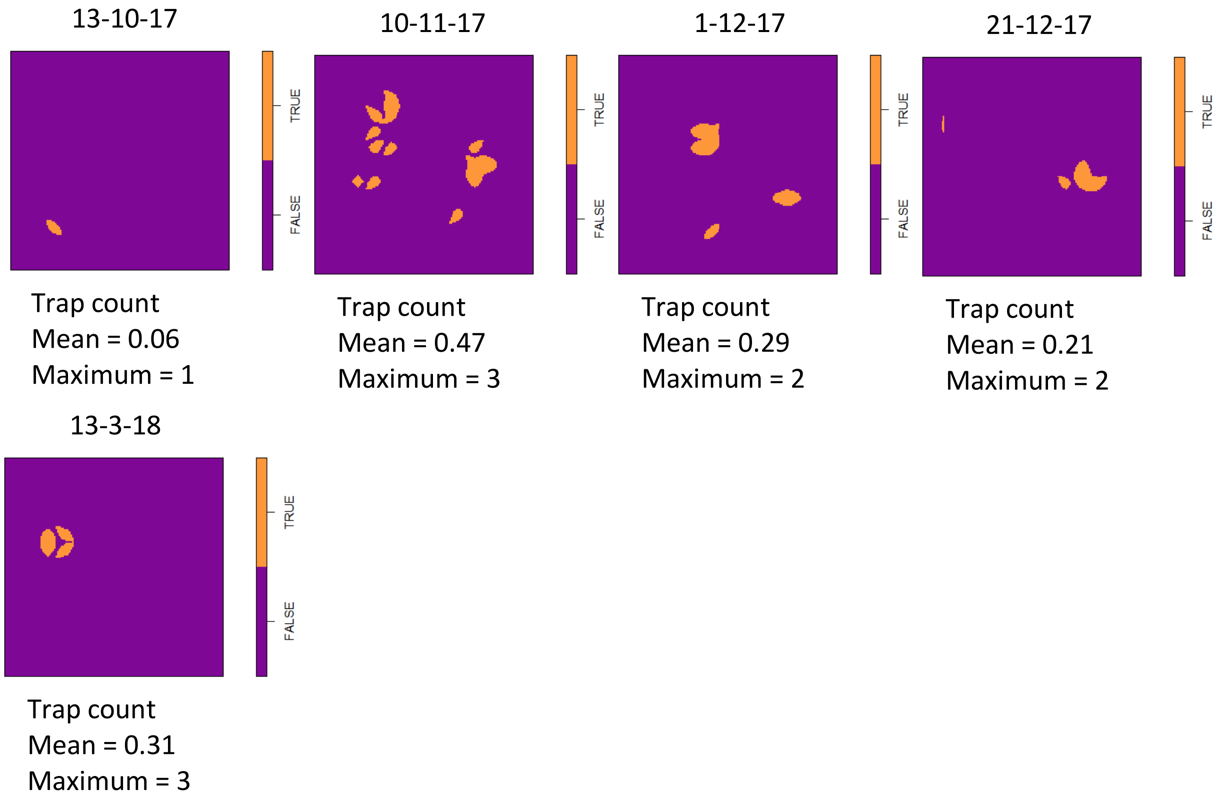

Figure S19. Visualized grid maps showing the location of domains of higher slug density identified by hotspot analysis of data collected from the rectangular grid of 100 refuge traps established at the Leic 1 field site during the 2017-18 field season. Data for each of the 5 assessments made at this site were analysed separately; grid squares coloured in amber indicate the location of discrete domains with significantly ($P<0.05$) higher numbers of slugs than would be expected if a random distribution is assumed. Mean trap count = mean number of slugs caught in the 100 traps in the sampling grid; Maximum trap count = largest number of slugs caught by an individual trap.

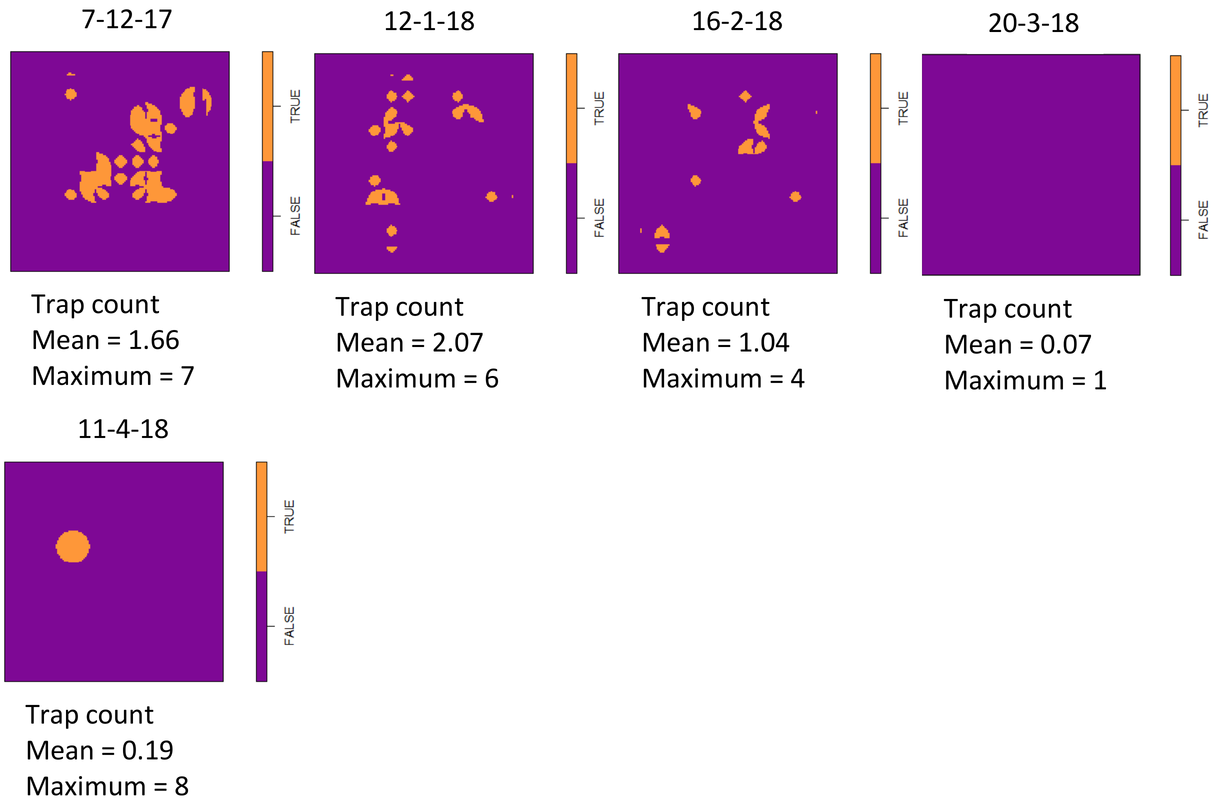

Figure S20. Visualized grid maps showing the location of domains of higher slug density identified by hotspot analysis of data collected from the rectangular grid of 100 refuge traps established at the Lancs 1 field site during the 2017-18 field season. Data for each of the 5 assessments made at this site were analysed separately; grid squares coloured in amber indicate the location of discrete domains with significantly ($P<0.05$) higher numbers of slugs than would be expected if a random distribution is assumed. Mean trap count = mean number of slugs caught in the 100 traps in the sampling grid; Maximum trap count = largest number of slugs caught by an individual trap.

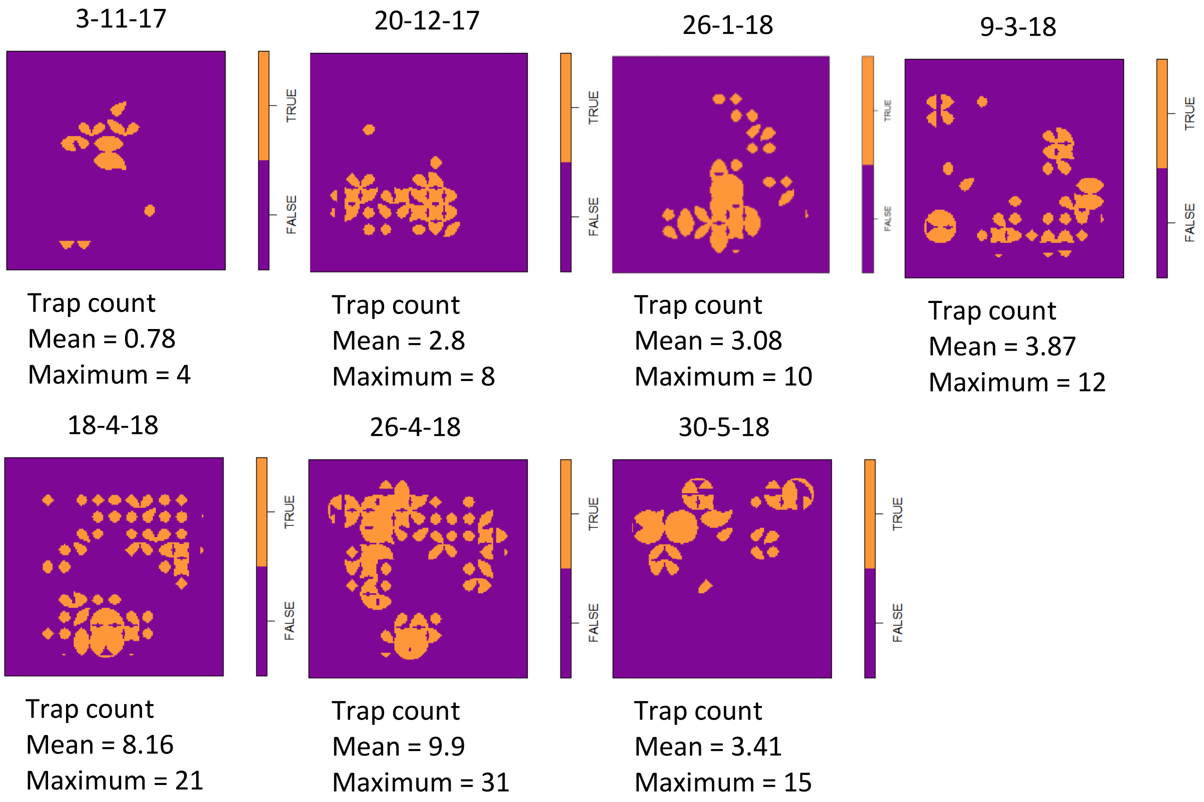

Figure S21. Visualized grid maps showing the location of domains of higher slug density identified by hotspot analysis of data collected from the rectangular grid of 100 refuge traps established at the Shrop 7 field site during the 2017-18 field season. Data for each of the 7 assessments made at this site were analysed separately; grid squares coloured in amber indicate the location of discrete domains with significantly ($P<0.05$) higher numbers of slugs than would be expected if a random distribution is assumed. Mean trap count = mean number of slugs caught in the 100 traps in the sampling grid; Maximum trap count = largest number of slugs caught by an individual trap.

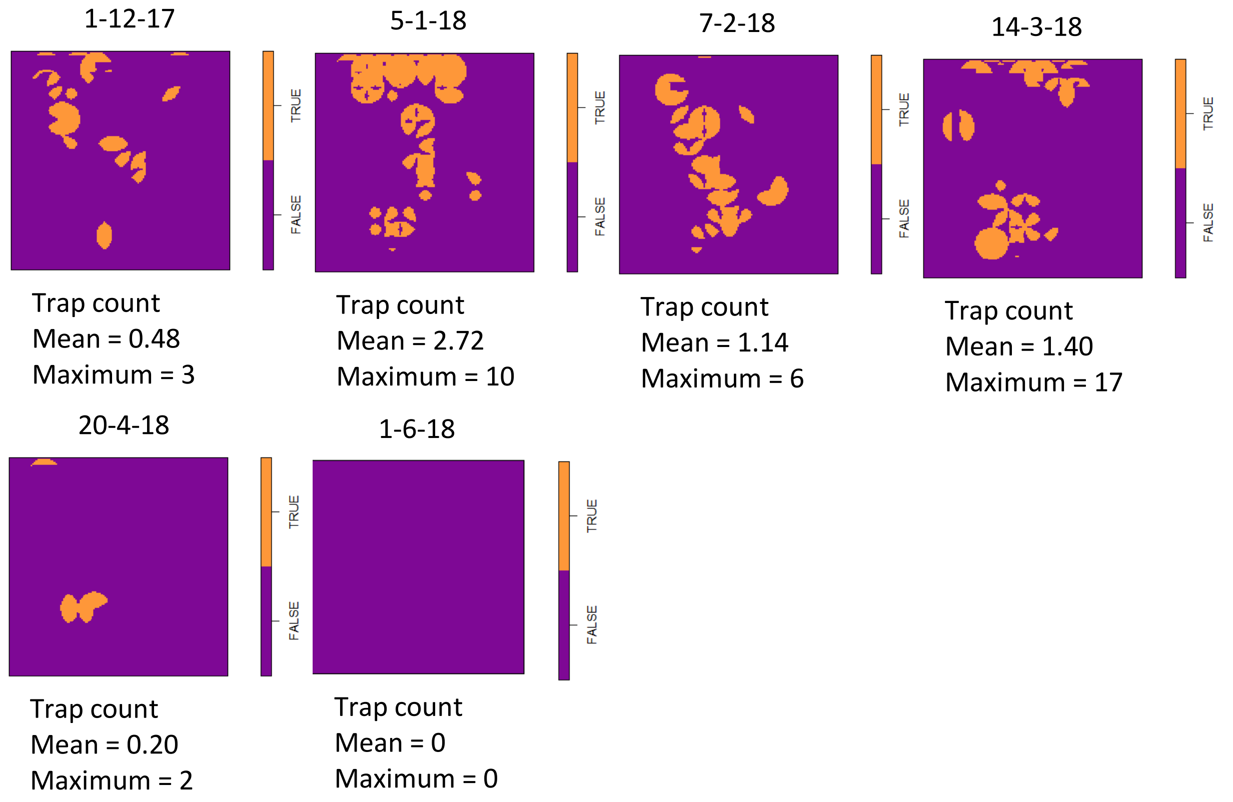

Figure S22. Visualized grid maps showing the location of domains of higher slug density identified by hotspot analysis of data collected from the rectangular grid of 100 refuge traps established at the Lincs 4 field site during the 2017-18 field season. Data for each of the 6 assessments made at this site were analysed separately; grid squares coloured in amber indicate the location of discrete domains with significantly ($P<0.05$) higher numbers of slugs than would be expected if a random distribution is assumed. Mean trap count = mean number of slugs caught in the 100 traps in the sampling grid; Maximum trap count = largest number of slugs caught by an individual trap.
