# Supplementary material for: Stability of Patches of Higher Population Density within the Heterogenous Distribution of the Gray Field Slug Deroceras reticulatum in Arable Fields in the UK
Source: Insects. 2020 Dec 25;12(1):9. doi: 10.3390/insects12010009 (PMC7823367; doi:10.3390/insects12010009)
Supplement: Supplementary file 1 [file insects-12-00009-s001.zip › supplementary-xml/Supplementary Table 1.pdf]

Table S1. The spatio-temporal stability of areas of higher slug density within a regular 10 × 10m (1ha) sampling grid established in the Shrop 1 field site in 2015/16. On each assessment date the number of slugs recorded in a surface refuge trap established at each trapping node, collectively formed a data matrix describing the distribution of slug activity across the grid. The data matrices recorded on different assessment dates were compared using a Mantel permutation's test to investigate consistent effects of trap location on number of slugs caught. All possible pairwise permutations of assessment dates were analysed to determine whether significant correlations between the pairs of matrices could be identified. Dates are the days on which individual assessments were made; Mean = mean trap catch on each assessment date; Max = maximum individual trap catch; results of Mantel permutation's tests (*r* and *p* - corrected to 2 decimal places in the table). Significant correlation between the pairs of matrices are highlighted in grey.

**Shrop 1**	**30/11/2015**	**14/12/2015**	**5/1/2016**	**15/1/2016**	**29/1/2016**	**16/2/2016**
**No. slugs trap^{-1}:**	**Mean** = 0.3<br>**Max** = 4	**Mean** = 0.7<br>**Max** = 3	**Mean** = 1.3<br>**Max** = 7	**Mean** = 0.5<br>**Max** = 5	**Mean** = 1.0<br>**Max** = 5	**Mean** = 0.4<br>**Max** = 5
	***r* value; *p* value**	***r* value; *p* value**	***r* value; *p* value**	***r* value; *p* value**	***r* value; *p* value**	***r* value; *p* value**
**14/12/2015**	0.16; <0.05					
**5/1/2016**	0.04; 0.76	0.05; 0.15				
**15/1/2016**	0.07; 0.92	0.01; 0.43	0.11; <0.05			
**29/1/2016**	0.06; 0.86	0.04; 0.17	0.24; <0.01	0.02; 0.50		
**16/2//2016**	0.00; 0.40	0.01; 0.44	0.08; 0.09	0.42; <0.001	0.06; 0.14	
**26/4/2016**	0.07; 0.14	0.03; 0.24	0.04; 0.73	0.02; 0.53	0.01; 0.53	0.06; 0.16

Table S2. The spatio-temporal stability of areas of higher slug density within a regular 10 × 10m (1ha) sampling grid established in the Shrop 2 field site in 2015/16. On each assessment date the number of slugs recorded in a surface refuge trap established at each trapping node, collectively formed a data matrix describing the distribution of slug activity across the grid. The data matrices recorded on different assessment dates were compared using a Mantel permutation's test to investigate consistent effects of trap location on number of slugs caught. All possible pairwise permutations of assessment dates were analysed to determine whether significant correlations between the pairs of matrices could be identified. Dates are the days on which individual assessments were made; Mean = mean trap catch on each assessment date; Max = maximum individual trap catch; results of Mantel permutation's tests (r and p - corrected to 2 decimal places in the table). Significant correlation between the pairs of matrices are highlighted in grey.

**Shrop 2**	**08/12/2015**	**22/12/2015**	**05/01/2016**	**11/01/2016**	**14/01/2016**	**18/01/2016**	**25/01/2016**	**29/01/2016**	**09/02/2016**	**12/02/2016**
**No slugs trap^{-1}:**	**Mean** = 1.2 **Max** = 4	**Mean** = 1.7 **Max** = 7	**Mean** = 3.0 **Max** = 10	**Mean** = 2.7 **Max** = 9	**Mean** = 2.3 **Max** = 15	**Mean** = 4 **Max** = 15	**Mean** = 2.5 **Max** = 19	**Mean** = 2.7 **Max** = 12	**Mean** = 2.5 **Max** = 13	**Mean** = 2.4 **Max** = 9
	***r* value; *p* value**	***r* value; *p* value**	***r* value; *p* value**	***r* value; *p* value**	***r* value; *p* value**	***r* value; *p* value**	***r* value; *p* value**	***r* value; *p* value**	***r* value; *p* value**	***r* value; *p* value**
**22/12/201**	0.01; 0.38									
**5/1/2016**	0.01; 0.33	0.07; 0.10								
**11/1/2016**	0.03; 0.70	0.00; 0.41	0.21; <0.01							
**14/1/2016**	0.05; 0.92	0.05; 0.14	0.31; <0.001	0.39; <0.001						
**18/1/2016**	0.00; 0.39	0.00; 0.42	0.35; <0.001	0.44; <0.001	0.54;< 0.001					
**25/1/2016**	0.02; 0.26	0.12; <0.05	0.30; <0.001	0.33; <0.001	0.52; <0.001	0.57; <0.001				
**29/1/2016**	0.06; 0.10	0.17; <0.05	0.07; 0.10	0.09; 0.086	0.14; <0.05	0.15; <0.05	0.32; <0.01			
**9/2/2016**	0.07; 0.98	0.01; 0.43	0.37; <0.01	0.29; <0.01	0.39; <0.01	0.37; <0.001	0.38; <0.001	0.19; <0.05		
**12/2/2016**	0.03; 0.78	0.01; 0.37	0.20; <0.01	0.09; 0.059	0.18; <0.05	0.24; <0.01	0.28; <0.001	0.11; 0.054	0.35; <0.001	
**26/4/2016**	0.02; 0.25	0.04; 0.19	0.20; <0.05	0.28; <0.001	0.41; <0.01	0.38; <0.01	0.37; <0.001	0.28; <0.01	0.33; <0.01	0.15; <0.05

Table S3. The spatio-temporal stability of areas of higher slug density within a regular 10 × 10m (1ha) sampling grid established in the Shrop 3 field site in 2015/16. On each assessment date the number of slugs recorded in a surface refuge trap established at each trapping node, collectively formed a data matrix describing the distribution of slug activity across the grid. The data matrices recorded on different assessment dates were compared using a Mantel permutation's test to investigate consistent effects of trap location on number of slugs caught. All possible pairwise permutations of assessment dates were analysed to determine whether significant correlations between the pairs of matrices could be identified. Dates are the days on which individual assessments were made; Mean = mean trap catch on each assessment date; Max = maximum individual trap catch; results of Mantel permutation's tests (*r* and *p* corrected to 2 decimal places in the table). Significant correlation between the pairs of matrices are highlighted in grey.

**Shrop 3**	**08/12/2015**	**18/12/2015**	**06/01/2016**	**20/01/2016**	**03/02/2016**	**18/02/2016**
**No. slugs trap^{-1}:**	**Mean** = 1.6 **Max** = 6	**Mean** = 1.6 **Max** = 8	**Mean** = 2.2 **Max** = 9	**Mean** = 1.6 **Max** = 8	**Mean** = 3 **Max** = 14	**Mean** = 4 **Max** = 12
	***r* value; *p* value**	***r* value; *p* value**	***r* value; *p* value**	***r* value; *p* value**	***r* value; *p* value**	***r* value; *p* value**
**18/12/2015**	0.15; <0.05					
**6/1/2016**	0.07; 0.10	0.00; 0.39				
**20/1/2016**	0.02; 0.30	0.03; 0.25	0.04; 0.69			
**3/2/2016**	0.17; <0.01	0.15; <0.05	0.04; 0.20	0.02; 0.60		
**18/2/2016**	0.14; <0.01	0.08; 0.08	0.05; 0.84	0.11; 0.035	0.33; <0.001	
**25/5/2016**	0.08; 0.97	0.03; 0.62	0.06; 0.88	0.03; 0.65	0.02; 0.57	0.00; 0.43

Table S4. The spatio-temporal stability of areas of higher slug density within a regular 10 × 10m (1ha) sampling grid established in the Shrop 4 field site in 2015/16. On each assessment date the number of slugs recorded in a surface refuge trap established at each trapping node, collectively formed a data matrix describing the distribution of slug activity across the grid. The data matrices recorded on different assessment dates were compared using a Mantel permutation's test to investigate consistent effects of trap location on number of slugs caught. All possible pairwise permutations of assessment dates were analysed to determine whether significant correlations between the pairs of matrices could be identified. Dates are the days on which individual assessments were made; Mean = mean trap catch on each assessment date; Max = maximum individual trap catch; results of Mantel permutation's tests (*r* and *p* - corrected to 2 decimal places in the table). Significant correlation between the pairs of matrices are highlighted in grey.

**Shrop 4**	**07/12/2015**	**18/12/2015**	**22/12/2015**	**06/01/2016**	**11/01/2016**	**14/01/2016**	**21/01/2016**	**26/01/2016**	**02/02/2016**	**18/02/2016**	**15/03/2016**	**02/05/2016**
**No slugs trap^{-1}:**	**Mean** = 3.0 **Max** = 9	**Mean** = 4.7 **Max** = 14	**Mean** = 4.7 **Max** = 14	**Mean** = 6.0 **Max** = 21	**Mean** = 6.2 **Max** = 27	**Mean** = 6.0 **Max** = 48	**Mean** = 6.9 **Max** = 49	**Mean** = 8.0 **Max** = 25	**Mean** = 6.5 **Max** = 23	**Mean** = 9.1 **Max** = 49	**Mean** = 18 **Max** = 73	**Mean** = 17.5 **Max** = 63
	***r* value; *p* value**	***r* value; *p* value**	***r* value; *p* value**	***r* value; *p* value**	***r* value; *p* value**	***r* value; *p* value**	***r* value; *p* value**	***r* value; *p* value**	***r* value; *p* value**	***r* value; *p* value**	***r* value; *p* value**	***r* value; *p* value**
**18/12/2015**	0.13; <0.05											
**22/12/2015**	0.03; 0.24	0.22; <0.001										
**6/1/2016**	0.02; 0.32	0.43; <0.001	0.28; <0.001									
**11/1/2016**	0.02; 0.33	0.32; <0.001	0.22; <0.001	0.50; <0.001								
**14/1/2016**	0.03; 0.62	0.36; <0.001	0.35; <0.001	0.53; <0.001	0.78; 0.001							
**21/1/2016**	0.03; 0.63	0.35; <0.001	0.26, <0.01	0.45; <0.001	0.73; 0.001	0.79; <0.001						
**26/1/2016**	0.001; 0.52	0.02; 0.30	0.01; 0.52	0.04; 0.23	0.00; 0.42	0.02; 0.59	0.01; 0.35					
**2/2/2016**	0.08; 0.97	0.19; <0.01	0.30; <0.001	0.31; <0.001	0.29; <0.001	0.43; <0.001	0.33; <0.001	0.08; 0.085				
**18/2/2016**	0.08; 0.96	0.26; <0.01	0.33; <0.001	0.35; <0.001	0.64; <0.001	0.72; <0.001	0.64; <0.001	0.02; 0.30	0.50; <0.001			
**15/3/2016**	0.08; 0.95	0.23; <0.01	0.28; <0.001	0.32; <0.001	0.57; <0.001	0.60; <0.001	0.62; <0.001	0.05; 0.19	0.32; <0.001	0.64; <0.001		
**2/5/2016**	0.08; 0.96	0.23; <0.01	0.28; <0.001	0.32; <0.001	0.57; <0.001	0.60; <0.001	0.62; <0.001	0.05; 0.20	0.32; <0.001	0.64; <0.001	0.07; 0.10	
**24/5/2016**	0.06; 0.13	0.14; <0.05	0.031; 0.24	0.16; <0.05	0.13; <0.05	0.20; <0.05	0.15; <0.05	0.02; 0.33	0.11; 0.06	0.07; 0.12	0.13; 0.05	0.36; <0.001

Table S5. The spatio-temporal stability of areas of higher slug density within a regular 10 × 10m (1ha) sampling grid established in the Shrop 5 field site in 2015/16. On each assessment date the number of slugs recorded in a surface refuge trap established at each trapping node, collectively formed a data matrix describing the distribution of slug activity across the grid. The data matrices recorded on different assessment dates were compared using a Mantel permutation's test to investigate consistent effects of trap location on number of slugs caught. All possible pairwise permutations of assessment dates were analysed to determine whether significant correlations between the pairs of matrices could be identified. Dates are the days on which individual assessments were made; Mean = mean trap catch on each assessment date; Max = maximum individual trap catch; results of Mantel permutation's tests (*r* and *p* - corrected to 2 decimal places in the table). Significant correlation between the pairs of matrices are highlighted in grey.

**Shrop 5**	**07/12/2015**	**17/12/2015**	**04/01/2016**	**19/01/2016**	**01/02/2016**	**16/02/2016**	**29/04/2016**
**No slugs trap^{-1}:**	**Mean =** 1.5<br>**Max =** 5	**Mean =** 1.7<br>**Max =** 6	**Mean =** 1.8<br>**Max =** 6	**Mean =** 1.3<br>**Max=** 6	**Mean =** 1.6<br>**Max =** 8	**Mean =** 0.2<br>**Max =** 2	**Mean =** 6.7<br>**Max =** 17
	***r* value; *p* value**	***r* value; *p* value**	***r* value; *p* value**	***r* value; *p* value**	***r* value; *p* value**	***r* value; *p* value**	***r* value; *p* value**
**17/12/2015**	0.15; <0.01						
**4/1/2016**	0.13; <0.05	0.09; <0.05					
**19/1/2016**	0.05; 0.83	0.09; 0.99	0.09; 0.99				
**1/2/2016**	0.01; 0.37	0.03; 0.73	0.01; 0.37	0.06; 0.88			
**16/2/2016**	0.04; 0.78	0.07; 0.94	0.00; 0.45	0.08; 0.11	0.04; 0.71		
**29/4/2016**	0.07; 0.95	0.07; 0.94	0.04; 0.20	0.09; 0.07	0.01; 0.37	0.01; 0.49	
**23/5/2016**	0.03; 0.26	0.03; 0.21	0.06; 0.13	0.15; <0.05	0.01; 0.34	0.11; 0.08	0.02; 0.28

Table S6. The spatio-temporal stability of areas of higher slug density within a regular 10 × 10m (1ha) sampling grid established in the Lancs 1 field site in 2016/17. On each assessment date the number of slugs recorded in a surface refuge trap established at each trapping node, collectively formed a data matrix describing the distribution of slug activity across the grid. The data matrices recorded on different assessment dates were compared using a Mantel permutation's test to investigate consistent effects of trap location on number of slugs caught. All possible pairwise permutations of assessment dates were analysed to determine whether significant correlations between the pairs of matrices could be identified. Dates are the days on which individual assessments were made; Mean = mean trap catch on each assessment date; Max = maximum individual trap catch; results of Mantel permutation's tests (r and p - corrected to 2 decimal places in the table). Significant correlation between the pairs of matrices are highlighted in grey.

**Lancs 1**	**20/10/2016**	**21/11/2016**	**21/12/2016**	**18/01/2017**	**08/02/2017**	**02/03/2017**	**22/03/2017**	**12/04/2017**	**10/05/2017**
**No slugs trap^{-1}:**	**Mean** = 0.2 **Max** = 2	**Mean** = 0.1 **Max** = 2	**Mean** = 0.3 **Max** = 2	**Mean** = 0.7 **Max** = 5	**Mean** = 0.4 **Max** = 3	**Mean** = 0.8 **Max** = 5	**Mean** = 0.9 **Max** = 5	**Mean** = 2 **Max** = 8	**Mean** = 4.6 **Max** = 24
	***r* value; *p* value**	***r* value; *p* value**	***r* value; *p* value**	***r* value; *p* value**	***r* value; *p* value**	***r* value; *p* value**	***r* value; *p* value**	***r* value; *p* value**	***r* value; *p* value**
**21/11/2016**	0.06; 0.19								
**21/12/2016**	0.27; <0.01	0.03; 0.57							
**18/1/2017**	0.02; 0.34	0.05; 0.69	0.15; <0.05						
**8/2/2017**	0.03; 0.65	0.05; 0.75	0.07; 0.13	0.15; <0.05					
**2/3/2017**	0.10; 0.08	0.10; 0.08	0.01; 0.33	0.06; 0.13	0.19; <0.01				
**22/3/2017**	0.01; 0.46	0.04; 0.22	0.05; 0.18	0.03; 0.24	0.04; 0.19	0.01; 0.50			
**12/4/2017**	0.03; 0.26	0.06; 0.13	0.00; 0.41	0.02; 0.27	0.02; 0.31	0.04; 0.80	0.14; <0.01		
**10/5/2017**	0.01; 0.45	0.04; 0.23	0.05; 0.19	0.03; 0.24	0.04; 0.19	0.01; 0.49	1.0; <0.001	0.14; <0.01	
**8/6/2017**	0.02; 0.27	0.06; 0.13	0.00; 0.42	0.02; 0.27	0.02; 0.30	0.04; 0.79	0.14; <0.01	1.0; <0.001	0.14; <0.01

Table S7. The spatio-temporal stability of areas of higher slug density within a regular 10 × 10m (1ha) sampling grid established in the Shrop 2 field site in 2016/17. On each assessment date the number of slugs recorded in a surface refuge trap established at each trapping node, collectively formed a data matrix describing the distribution of slug activity across the grid. The data matrices recorded on different assessment dates were compared using a Mantel permutation's test to investigate consistent effects of trap location on number of slugs caught. All possible pairwise permutations of assessment dates were analysed to determine whether significant correlations between the pairs of matrices could be identified. Dates are the days on which individual assessments were made; Mean = mean trap catch on each assessment date; Max = maximum individual trap catch; results of Mantel permutation's tests (r and p - corrected to 2 decimal places in the table). Significant correlation between the pairs of matrices are highlighted in grey.

**Shrop 2**	**05/09/2016**	**15/12/2016**	**16/01/2017**	**07/02/2017**	**07/03/2017**	**04/04/2017**	**02/05/2017**
**No slugs trap^{-1}:**	**Mean** = 0.6 **Max** = 3	**Mean** = 0.4 **Max** = 3	**Mean** = 0.4 **Max** = 4	**Mean** = 0.3 **Max** = 2	**Mean** = 0.5 **Max** = 3	**Mean** = 0.5 **Max** = 2	**Mean** = 0.2 **Max** = 2
	***r* value; *p* value**	***r* value; *p* value**	***r* value; *p* value**	***r* value; *p* value**	***r* value; *p* value**	***r* value; *p* value**	***r* value; *p* value**
**15/12/2016**	0.05; 0.78						
**16/1/2017**	0.04; 0.72	0.14; 0.05					
**7/2/2017**	0.10; 0.07	0.14; 0.04	0.17; <0.05				
**7/3/2017**	0.05; 0.17	0.11; 0.05	0.04; 0.73	0.02; 0.34			
**4/4/2017**	0.03; 0.75	0.07; 0.99	0.05; 0.15	0.03; 0.62	0.00; 0.42		
**2/5/2017**	0.01; 0.50	0.02; 0.56	0.03; 0.31	0.06; 0.19	0.02; 0.32	0.10; <0.05	
**31/5/2017**	0.06; 1.0	0.05; 0.56	0.02; 0.38	0.03; 0.60	0.03; 0.61	0.05; 0.22	0.01; 0.37

Table S8. The spatio-temporal stability of areas of higher slug density within a regular 10 × 10m (1ha) sampling grid established in the Shrop 5 field site in 2016/17. On each assessment date the number of slugs recorded in a surface refuge trap established at each trapping node, collectively formed a data matrix describing the distribution of slug activity across the grid. The data matrices recorded on different assessment dates were compared using a Mantel permutation's test to investigate consistent effects of trap location on number of slugs caught. All possible pairwise permutations of assessment dates were analysed to determine whether significant correlations between the pairs of matrices could be identified. Dates are the days on which individual assessments were made; Mean = mean trap catch on each assessment date; Max = maximum individual trap catch; results of Mantel permutation's tests (*r* and *p* - corrected to 2 decimal places in the table). Significant correlation between the pairs of matrices are highlighted in grey.

**Shrop 5**	**13/09/2016**	**18/10/2016**	**23/11/2016**	**20/12/2016**	**17/01/2017**	**10/02/2017**
**No slugs trap^{-1}:**	**Mean** = 0.5 **Max** = 5	**Mean** = 1.5 **Max** = 6	**Mean** = 2.3 **Max** = 7	**Mean** = 1.5 **Max** = 10	**Mean** = 0.8 **Max** = 6	**Mean** = 1.5 **Max** = 7
	***r* value; *p* value**	***r* value; *p* value**	***r* value; *p* value**	***r* value; *p* value**	***r* value; *p* value**	***r* value; *p* value**
**18/10/2016**	0.08; 0.10					
**23/11/2016**	0.01; 0.52	0.06; 0.11				
**20/12/2016**	0.07; 0.13	0.25; <0.01	0.04; 0.20			
**17/1/2017**	0.04; 0.19	0.05; 0.16	0.07; 0.10	0.01; 0.44		
**10/2/2017**	0.13; <0.05	0.00; 0.40	0.03; 0.71	0.13; <0.05	0.13; <0.05	
**28/2/2017**	0.18; <0.01	0.02; 0.57	0.02; 0.31	0.18; <0.05	0.10; 0.06	0.18; <0.01

Table S9. The spatio-temporal stability of areas of higher slug density within a regular 10 × 10m (1ha) sampling grid established in the Lincs 4 field site in 2017/18. On each assessment date the number of slugs recorded in a surface refuge trap established at each trapping node, collectively formed a data matrix describing the distribution of slug activity across the grid. The data matrices recorded on different assessment dates were compared using a Mantel permutation's test to investigate consistent effects of trap location on number of slugs caught. All possible pairwise permutations of assessment dates were analysed to determine whether significant correlations between the pairs of matrices could be identified. Dates are the days on which individual assessments were made; Mean = mean trap catch on each assessment date; Max = maximum individual trap catch; results of Mantel permutation's tests (*r* and *p* - corrected to 2 decimal places in the table).

**Lincs 4**	**01/12/2017**	**05/01/2018**	**07/02/2018**	**14/03/2018**	**20/04/2018**
**No slugs trap^{-1}:**	**Mean =** 0.5 **Max =** 3	**Mean =** 2.7 **Max =** 10	**Mean =** 1.1 **Max =** 6	**Mean =** 1.4 **Max =** 17	**Mean =** 0.2 **Max =** 2
	***r* value; *p* value**	***r* value; *p* value**	***r* value; *p* value**		***r* value; *p* value**
**5/1/2018**	0.01; 0.53				
**7/2/2018**	0.02; 0.59	0.09; 0.06			
**14/3/2018**	0.03; 0.63	0.01; 0.32	0.08; 0.12		
**20/4/2018**	0.08; 0.92	0.04; 0.75	0.09; 0.95	0.10; 0.10	
**1/6/2018**	No slugs	No slugs	No slugs	No slugs	No slugs

Table S10. The spatio-temporal stability of areas of higher slug density within a regular 10 × 10m (1ha) sampling grid established in the Shrop 2 field site in 2017/18. On each assessment date the number of slugs recorded in a surface refuge trap established at each trapping node, collectively formed a data matrix describing the distribution of slug activity across the grid. The data matrices recorded on different assessment dates were compared using a Mantel permutation's test to investigate consistent effects of trap location on number of slugs caught. All possible pairwise permutations of assessment dates were analysed to determine whether significant correlations between the pairs of matrices could be identified. Dates are the days on which individual assessments were made; Mean = mean trap catch on each assessment date; Max = maximum individual trap catch; results of Mantel permutation's tests (*r* and *p* - corrected to 2 decimal places in the table). Significant correlation between the pairs of matrices are highlighted in grey.

**Shrop 2**	**28/09/2017**	**20/10/2017**	**20/12/2017**	**26/01/2018**	**08/03/2018**	**21/03/2018**	**15/05/2018**
**No slugs trap^{-1}:**	**Mean** = 0.05 **Max** = 1	**Mean** = 0.2 **Max** = 3	**Mean** = 0.6 **Max** = 4	**Mean** = 1.2 **Max** = 6	**Mean** = 1 **Max** = 7	**Mean** = 1 **Max** = 7	**Mean** = 0.04 **Max** = 1
	***r* value; *p* value**	***r* value; *p* value**	***r* value; *p* value**	***r* value; *p* value**	***r* value; *p* value**	***r* value; *p* value**	***r* value; *p* value**
**20/10/2017**	0.04; 0.13						
**20/12/2017**	0.02; 0.45	0.01; 0.48					
**26/1/2018**	0.00; 0.34	0.01; 0.49	0.09; 0.05				
**8/3/2018**	0.03; 0.42	0.05; 0.77	0.21; <0.01	0.17; <0.01			
**21/3/2018**	0.03; 0.53	0.09; 0.10	0.17; < 0.05	0.16; <0.05	0.09; 0.09		
**15/5/2018**	0.03; 0.79	0.06; 0.93	0.02; 0.31	0.05; 0.15	0.04; 0.23	0.06; 0.72	
**30/5/2018**	0.05; 1.0	0.02; 0.57	0.08; 0.98	0.02; 0.49	0.04; 0.66	0.01; 0.47	0.05; 0.56

Table S11. The spatio-temporal stability of areas of higher slug density within a regular 10 × 10m (1ha) sampling grid established in the Shrop 7 field site in 2017/18. On each assessment date the number of slugs recorded in a surface refuge trap established at each trapping node, collectively formed a data matrix describing the distribution of slug activity across the grid. The data matrices recorded on different assessment dates were compared using a Mantel permutation's test to investigate consistent effects of trap location on number of slugs caught. All possible pairwise permutations of assessment dates were analysed to determine whether significant correlations between the pairs of matrices could be identified. Dates are the days on which individual assessments were made; Mean = mean trap catch on each assessment date; Max = maximum individual trap catch; results of Mantel permutation's tests (*r* and *p* - corrected to 2 decimal places in the table). Significant correlation between the pairs of matrices are highlighted in grey.

**Shrop 7**	**03/11/2017**	**20/12/2017**	**09/03/2018**	**18/04/2018**	**26/04/2018**
**No slugs trap^{-1}:**	**Mean** = 0.8 **Max** = 4	**Mean** = 2.8 **Max** = 8	**Mean** = 3.9 **Max** = 12	**Mean** = 8.2 **Max** = 21	**Mean** = 9.9 **Max** = 31
	***r* value; *p* value**	***r* value; *p* value**	***r* value; *p* value**	***r* value; *p* value**	***r* value; *p* value**
**20/12/2017**	0.01; 0.38				
**9/3/2018**	0.00; 0.43	0.04; 0.83			
**18/4/2018**	0.05; 0.17	0.05; 0.14	0.01; 0.36		
**26/4/2018**	0.02; 0.29	0.05; 0.85	0.06; 0.11	0.25; <0.001	
**30/5/2018**	0.06; 0.88	0.02; 0.66	0.06; 0.88	0.06; 0.89	0.15; <0.05
